# Epigenome-wide association study of DNA methylation in maternal blood leukocytes with BMI in pregnancy and gestational weight gain

**DOI:** 10.1038/s41366-024-01458-x

**Published:** 2024-01-13

**Authors:** J. O. Opsahl, N. Fragoso-Bargas, Y. Lee, E. Ø. Carlsen, N. Lekanova, E. Qvigstad, L. Sletner, A. K. Jenum, S. Lee-Ødegård, R. B. Prasad, K. I. Birkeland, G-H. Moen, C. Sommer

**Affiliations:** 1 Institute of Clinical Medicine, Faculty of Medicine, University of Oslo, Oslo, Norway; 2 Department of Endocrinology, Morbid Obesity and Preventive Medicine, Oslo University Hospital, Oslo, Norway; 3 Centre for Fertility and Health, Norwegian Institute of Public Health, Oslo, Norway; 4 Department of Biosciences, The Faculty of Mathematics and Natural Sciences, University of Oslo, Oslo, Norway; 5 Department of Pediatric and Adolescents Medicine, Akershus University Hospital, Lørenskog, Norway; 6 General Practice Research Unit, Department of General Practice, Institute of Health and Society, University of Oslo, Oslo, Norway; 7 Department of Nutrition, Institute of Basic Medical Sciences, Faculty of Medicine, University of Oslo, Oslo, Norway; 8 Lund University Diabetes Centre, Malmö, Sweden; 9 Institute for Molecular Medicine Finland (FIMM), University of Helsinki, Helsinki, Finland; 10 Department of Transplantation Medicine, Oslo University Hospital, Oslo, Norway; 11 The University of Queensland Diamantina Institute, The University of Queensland, Woolloongabba, QLD 4102 Australia; 12 K.G. Jebsen Center for Genetic Epidemiology, Department of Public Health and Nursing, NTNU, Norwegian University of Science and Technology, Trondheim, Norway; 13 Population Health Science, Bristol Medical School, University of Bristol, Bristol, UK; 14 Institute of Molecular Biosciences, The University of Queensland, Brisbane, QLD Australia

**Keywords:** Risk factors, Epidemiology, Obesity

## Abstract

**Objectives:**

We aimed to discover CpG sites with differential DNA methylation in peripheral blood leukocytes associated with body mass index (BMI) in pregnancy and gestational weight gain (GWG) in women of European and South Asian ancestry. Furthermore, we aimed to investigate how the identified sites were associated with methylation quantitative trait loci, gene ontology, and cardiometabolic parameters.

**Methods:**

In the Epigenetics in pregnancy (EPIPREG) sample we quantified maternal DNA methylation in peripheral blood leukocytes in gestational week 28 with Illumina’s MethylationEPIC BeadChip. In women with European (*n* = 303) and South Asian (*n* = 164) ancestry, we performed an epigenome-wide association study of BMI in gestational week 28 and GWG between gestational weeks 15 and 28 using a meta-analysis approach. Replication was performed in the Norwegian Mother, Father, and Child Cohort Study, the Study of Assisted Reproductive Technologies (MoBa-START) (*n* = 877, mainly European/Norwegian).

**Results:**

We identified one CpG site significantly associated with GWG (*p* 5.8 × 10−8) and five CpG sites associated with BMI at gestational week 28 (*p* from 4.0 × 10–8 to 2.1 × 10–10). Of these, we were able to replicate three in MoBa-START; cg02786370, cg19758958 and cg10472537. Two sites are located in genes previously associated with blood pressure and BMI. DNA methylation at the three replicated CpG sites were associated with levels of blood pressure, lipids and glucose in EPIPREG (*p* from 1.2 × 10^−8^ to 0.04).

**Conclusions:**

We identified five CpG sites associated with BMI at gestational week 28, and one with GWG. Three of the sites were replicated in an independent cohort. Several genetic variants were associated with DNA methylation at cg02786379 and cg16733643 suggesting a genetic component influencing differential methylation. The identified CpG sites were associated with cardiometabolic traits.

**ClinicalTrials.gov registration no:**

Not applicable

## Introduction

Women who have overweight or obesity in pregnancy, or have excessive gestational weight gain (GWG), are at increased risk of pregnancy-related complications such as pre-eclampsia, gestational diabetes mellitus (GDM), and of having offspring that are large for gestational age [1–3]. Overweight, obesity and excessive GWG also increases the risk of developing type 2 diabetes mellitus [4, 5] and cardiovascular disease [6, 7] later in life for both the mother and her offspring.

Several genetic variants associated with body mass index (BMI) have also been associated with metabolic complications of obesity, such as type 2 diabetes mellitus [8]. However, genetic variation explains only parts of the risk for obesity-related metabolic complications, wherein epigenetics are thought to influence gene expression and thereby downstream disease outcomes [9, 10].

There is an increasing interest in studies of how environmental factors influence epigenetic signatures such as DNA methylation and thereby modify gene expression. Epidemiological studies of non-pregnant populations have identified several CpG sites across the genome with differential DNA methylation associated with BMI [9–11]. A few epigenome wide association studies (EWAS) of DNA methylation in offspring tissues and placenta in association with maternal body weight have been conducted [12]. To the best of our knowledge, there are currently no EWAS in maternal peripheral blood leukocytes of BMI in pregnancy or GWG.

Differences in allele frequencies and linkage disequilibrium across ancestry may influence the risk of developing different diseases [13, 14]. The interplay between genetics and environmental factors may explain why some develop disease and not others [15]. Although this interplay could lead to differences by ancestry, combining populations of different ancestries with genetic and environmental differences (such as different lifestyle habits) may help in the discovery of CpG sites with robust DNA methylation associated with lifestyle factors across ancestry and reduce the risk of false positives.

We hypothesize that high body mass index and excess weight gain in pregnancy is associated with DNA methylation of CpG sites. Specifically, we aim to examine if BMI in gestational week 28 and GWG in pregnancy in European and South Asian women were associated with DNA methylation levels in peripheral blood leukocytes. Further, we aimed to examine whether the identified CpG sites were associated also with methylation quantitative trait loci (mQTL), gene ontology, and cardiometabolic parameters.

## Methods

### Study population

STORK Groruddalen is a population-based cohort that included 823 healthy women in early pregnancy attending three public mother-child health clinics for antenatal care from 2008 to 2010 in the multi-ethnic area of Groruddalen, Oslo, Norway [16]. Women were eligible if they: (1) lived in the study districts; (2) planned to give birth at one of two study hospitals; (3) were <20 weeks pregnant; (4) could communicate in Norwegian or any of the eight translated languages; and (5) were able to give informed consent. Women with pre-gestational diabetes, or in need of intensive hospital follow-up during pregnancy, were excluded. The participation rate was 74%, 73% for South Asian women and 81.5% for European women [16].

In the EPIPREG (“Epigenetics in pregnancy”) sample we quantified maternal DNA methylation in peripheral blood leukocytes of nearly all European (*n* = 312 (87.2%)) and South Asian women (*n* = 168 (87.2%)) attending the second visit in the STORK Groruddalen cohort study [17].

The study, including the GWAS/EWAS analyses, has been approved by the Norwegian Regional Committee for Medical Health Research Ethics South/East, with reference number: (2015/1035). We obtained written informed consent from all participants before any study-related procedure.

### Body weight measurements and questionnaire data

Information on age, self-reported ethnicity, parity, smoking status and pre-pregnancy weight was collected, using an interview-administered questionnaire [16]. Body height was measured by trained study personnel at mean gestational week 15 ± 3 (weeks) with a fixed stadiometer. Smoking status and body weight, total body fat percentage, and truncal fat percentage were measured at gestational weeks 15 ± 3 and 28 ± 2 (weeks) using bioelectrical impedance (Tanita-Weight BC-418 MA) [16]. Pre-pregnancy BMI was calculated using the self-reported pre-pregnancy weight, and mid-gestational BMI was calculated using body weight measured in gestational week 28 ± 2, both divided by the square of the body height in meters. GWG (kg) was calculated by subtracting the measured body weight at gestational week 15 from that at gestational week 28.

### Cardiometabolic parameters

Systolic and diastolic blood pressure were measured in gestational weeks 15 ± 3 and 28 ± 2 using Omron HEM-700-E M6 Comfort [18]. The measurement was repeated three times, and the mean of the last two readings was used for analysis [18].

Fasting venous blood samples were drawn at gestational week 28 ± 2. The procedures for measuring or calculating the following parameters have been described in detail elsewhere: fasting plasma glucose [16], fasting insulin, c-peptide, homeostasis model assessment of beta-cell function (HOMA-B), and homeostatic model assessment for insulin resistance (HOMA-IR) [19], glycated hemoglobin (HbA1c) [20], fasting triglycerides, low-density lipoprotein (LDL), high-density lipoprotein (HDL), total cholesterol [21] and leptin [22]. The women underwent a 75 g oral glucose tolerance test at gestational week 28 ± 2 and the glucose level were measured on-site in venous EDTA blood samples (HemoCue, Angelholm, Sweden) [16]. During data-collection, women with fasting glucose ≥ 7.0 mmol/L or 2-h values ≥ 9 mmol/L were referred to follow-ups in secondary care according to current recommendations at the time; World Health Organization (WHO) 1999 criteria [16, 23]. Women with 2-h values of 7.8–8.9 mmol/L were given lifestyle advice and referred to follow-up in primary care [16]. For analytic purposes in this paper, we re-classified GDM status based on the WHO 2013 criteria: fasting plasma glucose 5.1–6.9 mmol/L and/or blood glucose 8.5–11.0 mmol/L 2 h after the oral glucose tolerance test [24].

### DNA isolation, DNA methylation, and genotyping

DNA was extracted using a salting out procedure [25], described previously [17]. DNA samples underwent bisulfite conversion using EZ DNA MethylationTM Kit (Zymo Research, Tustin, CA, USA) before being added onto Illumina’s MethylationEPIC (EPIC) BeadChip (San Diego, CA, USA) for analysis using Illumina’s GenomeStudio Software. All samples were placed randomly across chips to remove technical variation. Subsequently, the *meffil* [26] R package was used for (1) quality control; probes with detection *p*-value < 0.01, a bead count <3, sex mismatch >5 SD or genotype mismatch (*n* = 7 samples) were removed, (2) functional normalization, which return normalized data adjusted for batch effects (slide, row, column and 10 principal components), and (3) calculation of DNA methylation levels, represented as a beta (β) value of the fluorescent intensity ratio ranging from 0 (not methylated) to 1 (completely methylated). A total of 844,951 probes remained for the subsequent analysis. A total of 480 samples were available for subsequent analyses (312 samples of European ancestry and 168 of South Asian ancestry). DNA samples from 30 women were selected for technical replication analysis using pyrosequencing [17]. A total of 303 women of European ancestry and 164 women of South Asian ancestry had data on BMI at gestational week 28 ± 2 available for EWAS (Supplementary file 1, Supplementary Fig. 1).

DNA samples were genotyped using the Illumina Infinium CoreExome chip (San Diego, CA, USA) and the Illumina iScan software (San Diego, CA, USA) by the Department of Clinical Sciences, Clinical Research Centre, Lund University, Malmö, Sweden. Quality control and conversion to GWAS data were performed using the PLINK 1.9 software package [27]. European and South Asian ancestry was defined by informative principal component analysis based on the variance-standardized relationship matrix generated. Ancestry from principal components corresponded perfectly with self-reported ethnicity from the interview-administered questionnaire [17]. We excluded samples with low quality or low concentration, low call rates (< 95%), extreme heterozygosity (> mean ± (3x standard deviations[SD])|, *n* = 1), mismatched gender (*n* = 24, indicative of low quality and not true gender mismatch since all participants were pregnant women) or cryptic relatedness [one woman (chosen at random) from each related pair, defined as genome-wide identity by descent (IBD) > 0.185 (*n* = 6)]. After quality control, *n* = 293,914 variants were left for imputation. A total of 298 women of European ancestry (98.3%) and 138 women of South Asian ancestry (84.1%) passed the quality control and were available for mQTL analysis (Supplementary file 1, Supplementary Fig. 1).

### Statistics

Statistical analyses were performed using R v.3.6.0 [28]. Beta-values were logit-transformed to *M*-values, and 212 CpGs were subsequently removed because of the resulting infinite values. For the EWAS analysis of GWG and BMI in pregnancy, linear regression analyses were performed using the R package “*limma”[*29], with M-values as the dependent variable. Correction for batch effect is described in the section of *DNA isolation, DNA methylation, and genotyping*. Covariates adjusted for in the analyses were age, smoking status (current smoker, smoked three months before pregnancy, former smoker, and never smoker), and cell type composition was estimated with the Houseman algorithm [30] with the R-package “*meffil*” [26]. GWG was in addition adjusted for BMI in gestational week 15. We first performed the EWAS of BMI and GWG in the women of European and South Asian ancestry separately, followed by fixed-effects meta-analyses using METAL [31]. We removed CpG sites with very low variance CpG sites were determined to have very low variance if they had a 1% range in beta values (DNA methylation level ranging from 0 to 1) between the 10th and 90th percentile based on the formula by Edgar et al. [32], and ended up with a total of 806 236 CpG sites. Using the Bonferroni method to correct for multiple testing, CpG sites from the EWAS were considered statistically significant if they reached a *P*-value of 0.05/806 236 = 6 × 10^−8^. The results were visualized using the QQman package v.0.1.8 [33].

We attempted replication and report all sites with a false discovery rate (FDR) of <5% in Supplementary file 1 since Bonferroni correction is conservative.

### Replication in an independent cohort

The Norwegian Mother, Father and Child Cohort Study (MoBa) is a nationwide Norwegian pregnancy cohort study that between 1998 and 2008 recruited approximately 95,000 mothers, 75,000 fathers, and 114,000 children [34]. The cohort and data collection have been described in detail previously [34–36]. In a substudy of MoBa, the Study of Assisted Reproductive Technology (MoBa-START), DNA methylation levels were measured (and obtained) using the EPIC array in maternal peripheral blood drawn around gestational week 18 [37]. We focused on 877 samples from women of European ancestry who conceived naturally and were available for EWAS of BMI [37]. Further details on the MoBa questionnaires, sample collection, and quality control are presented in Supplementary File 1.

In the MoBa-START cohort, we performed linear regressions of self-reported BMI at gestational week 30 on DNA methylation in maternal peripheral blood leukocytes, with adjustment for maternal age at the time of birth (continuous), maternal smoking during pregnancy (never, former, quit before the 18th week of gestation, or continued smoking after the 18th week of gestation), and cell composition, estimated by FlowSorted.Blood.EPIC (https://github.com/immunomethylomics/FlowSorted.BloodExtended.EPIC).

### CpG sites for further analyses

From the EWAS of BMI using a meta-analysis approach, we further explored CpG sites (*p* < 6 × 10^−8^) that were replicated in MoBa-START (*p* < 0.05). Since DNA methylation was quantified in week 18 in MoBa-START, before the gestational weight gain, MoBa-START was considered inappropriate for replication of the identified GWG related CpG site due to differences in the study design. Despite not having a replication cohort, we further explored identified CpG sites from the EWAS of GWG (*p* < 6 × 10^−8^). These sites are referred to as BMI or GWG related CpG sites. The BMI or GWG related CpG sites were further pursued for consistency across timepoints (pre-pregnancy BMI, *p* < 0.05), cardiometabolic parameters (see abowe, *p* < 0.05) and covariates (age, smoking, gestational week, parity and cell type*, p* < 0.05), associations with genotype (see below), pathways (see below) and look-up in databases (see below).

### Association between replicated CpG sites and genotype (mQTL)

We performed linear regression separately in Europeans and South Asians using the R package GEM [38], adjusted for blood cell composition, age and smoking. BMI or GWG related CpG sites were queried, and we used genotypes post-imputation. *Cis-*mQTLs were defined as positioned <± 500,000 base pairs from the DNA methylation site, if further away within the same chromosome classified as *trans-*mQTLs. We report associations at *p*-value < 5 × 10^−8^. To prune the mQTLs, we used the SNPclip Tool in LDlink [39] to examine linkage disequilibrium for the identified genetic variants for Europeans and South Asians separately. The thresholds were set to the default of R^2^ 0.1 and the minor allele frequency of 0.01. As reference populations, we used Utah Residents from North and West Europe (CEU) for Europeans [39], and Punjabi from Lahore, Pakistan (PJL), Sri Lankan Tamil from the UK (STU), and Indian Telugu from the UK (ITU) for South Asians [39].

We used Phenoscanner v.2 [40] to identify phenotypes associated with genetic variants from the mQTL analysis, with *p* < 0.001 as significance threshold. We used the rs-number of the most significant gene variant from the linkage disequilibrium analysis (R^2^ = 0.9).

### Pathway enrichment analysis

Enrichment analyses were performed by first mapping the BMI or GWG related CpG sites (separately) to their nearest gene using the genoma database (https://genoma.io) [41]. The genes were then subjected to pathway enrichment analysis using hypergeometric tests for overlap with 50 well-established biological pathways obtained from MSigDB (“Hallmark pathways”) [42]. Gene ontology (GO) of molecular function, biological process, and cellular component, as well as the Kyoto Encyclopedia of Genes and Genome (KEGG), were searched to identify potential pathways. Identified pathways with a FDR of 5% were considered statistically significant.

### Lookups in databases

We queried the replicated BMI or GWG related CpG sites in the goDMC-database (http://meQTLdb.godmc.org.uk/), EWAS catalog (http://ewascatalog.org/), EWAS atlas (https://ngdc.cncb.ac.cn/ewas/atlas) and Phenoscanner v.2 [40]. In the Phenoscanner, we used chromosome numbers and positions of the CpG sites. Results with a *p*-values < 0.05 were considered statistically significant.

## Results

### Sample characteristics

The characteristics of the 467 study participants with available BMI at gestational week 28 ± 2 are presented in Table 1, stratified by ancestry: 303 women of European ancestry and 164 women of South Asian ancestry.

**Table 1:** Characteristics of the study participants in gestational week 28 ± 2 (otherwise stated).

	Europeans	South Asians
Participants, *n*	303	164
Age in whole years, mean (SD)	30.6 (4.5)	28.7 (4.5)
Height in cm, mean (SD)	167.4 (5.7)	159.8 (5.8)
Pre-pregnancy BMI (kg/m^2^), mean (SD)	25.2 (4.8)	24.4 (4.1)
Body mass index (kg/m^2^), mean (SD)	27.7 (4.8)	26.9 (4.1)
Gestational weight gain in kg, mean (SD)	14.5 (6.4)	12.6 (5.6)
Total fat mass percentage, median [IQR]	29.5 [28.4, 30.6]	26.3 [25.0, 27.6]
Truncal fat mass percentage, median [IQR]	15.8 [15.2, 16.4]	14.0 [13.2, 14.9]
Gestational diabetes mellitus, *n* (%)	73 (23.9)	68 (41.5)
HbA1c, mmol/mol (SD)	32 (0.3)	34 (0.3)
Serum fasting glucose, mmol/L (SD)	4.4 (0.5)	4.5 (0.5)
Serum fasting insulin, pmol/L, median [IQR]	48.00 [33.0, 70.00]	72.00 [57.0, 102.5]
Serum fasting C-peptide, pmol/L, median [IQR]	708.0 [558.0, 900.0]	855.5 [688.8, 1078.2]
HOMA-IR, percentage, mean (SD)	1.5 [1.2, 1.9]	1.8 [1.5, 2.3]
HOMA-B, percentage, median [IQR]	173.5 [151.3, 199.5]	179.6 [154.9, 207.9]
Serum fasting total cholesterol, mmol/L, mean (SD)	6.4 (1.1)	6.0 (1.0)
Serum fasting HDL, mmol/L, mean (SD)	1.9 (0.4)	1.9 (0.4)
Serum fasting LDL, mmol/L, mean (SD)	3.7 (1.0)	3.3 (0.9)
Serum fasting TAG, mmol/L, mean (SD)	2.0 (0.7)	2.0 (0.6)
Leptin, µg/L, median [IQR]	1.6, [1.0, 2.5]	2.3 [1.5, 3.3]
Systolic blood pressure (mmHg), mean (SD)	106.9 (9.6)	101.2 (8.7)
Diastolic blood pressure (mmHg), mean (SD)	68 (7.1)	66 (7.0)
Nulliparous, *n* (%)	156 (51.5)	66 (40.2)
Smoking status, *n* (%)		
Current	19 (6.2)	0 (0.0)
3 months pre-pregnancy	79 (25.9)	2 (1.2)
Former	87 (28.5)	10 (6.1)
Never	120 (39.3)	152 (92.7)

Data are mean (SD) for normally distributed variables and median [IQR] for non-normal variables.

Data are mean (95% CI) for normally distributed variables and median [IQR] for non-normal variables.

*CI* confidence interval, *IQR* interquartile range, *BMI* body mass index, *HbA1c* glycosylated hemoglobin A1c, *HOMA-IR* homeostatic model assessment for insulin resistance, *HOMA-B* homeostasis model assessment of beta-cell function, *HDL* high-density lipoprotein, *LDL* low-density lipoprotein, *TAG* triacylglycerol.

### Differentially methylated positions

**Fig. 1:**
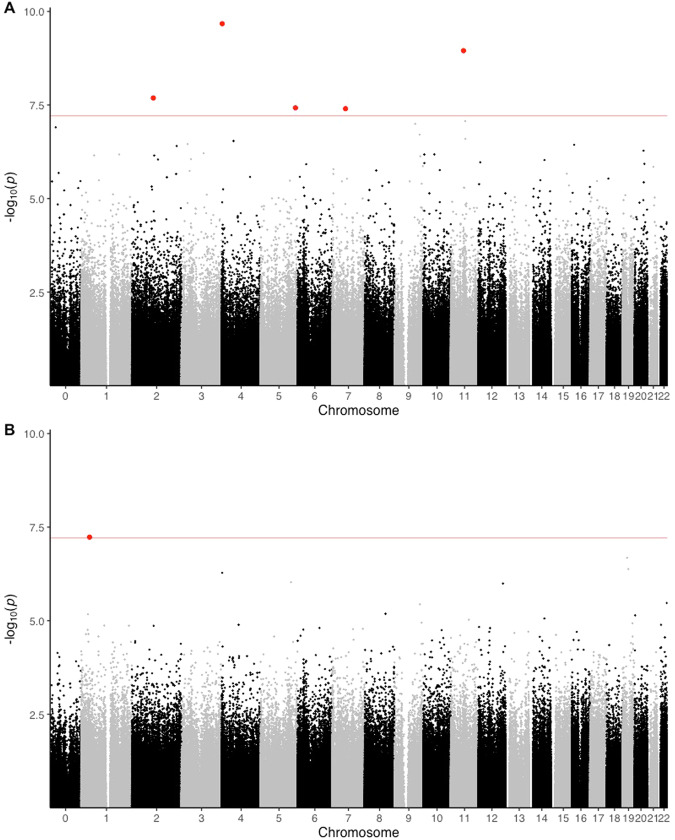
Manhattan plot. Epigenome wide association study (EWAS) of body mass index (BMI) and DNA methylation of maternal peripheral blood leukocytes drawn in gestational week 28 ± 2 in women of European (**A**) and South Asian (**B**) ancestry. *X*-axis: chromosome position in ascending order from 1 to the left to *X* on the right, *y*-axis: −log_10_*p-*value, red line: Bonferroni-corrected *p*-value, blue line: FDR 5%, Red dots: CpG sites significantly associated with BMI using Bonferroni correction, blue dots: CpG sites significantly associated with the BMI after correction for FDR < 0.05.

A total of 806,236 CpG sites were included in the EWASs of BMI and GWG (Fig. 1). The EWAS of BMI in women of European ancestry returned two significant CpG sites after Bonferroni correction (Table 2). The meta-analysis of BMI identified five CpG sites that were significant after Bonferroni correction (Table 2, Fig. 1). We found no evidence of systematic inflation in the EWAS of BMI of South Asian ancestry (*λ* = 0.933), however, some systematic inflation was observed in our analysis of BMI of European ancestry (*λ* = 1.318). For the EWAS of BMI, the cross-ancestry meta-analysis approach showed some systemic inflation (*λ* = 1.293).

**Table 2:** **CpG sites** from EWAS of body mass index and gestational weight gain significant after Bonferroni correction.

CpG-site	Chromosome	Position	Location in relation to gene	β	*SE*	*p*-value	Associated gene+	Gene summary **
*EWAS of body mass index, meta-analysis approach*								
cg02786370*	4	2,747,928	Open sea	−0.02	0.0031	2.12 × 10^−10^	*TNIP2*	NCBI gene ID 79155. Encodes a protein that inhibits NfkappaB-activation, is involved in MAP/ERK-signaling pathway in some cells, and could be involved in endothelial cell apoptosis.
Cg19758958*	11	62,319,222	Open sea	−0.01	0.0018	1.12 × 10^−9^	*Promoter region of AHNAC*	NCBI gene ID 79026. Encodes a structural scaffold protein that may play a role in blood-brain barrier formation, cell structure and migration, cardiac calcium channel regulation, and tumor metastasis
cg10472537*	2	105,348,800	Open sea	−0.01	0.002	2.05 × 10^−8^	*Unannotated*	
cg16444328	5	171,279,429	Open sea	−0.01	0.0015	3.76 × 10^−8^	*Unannotated*	
cg24911837	7	65,227,864	North shore	−0.01	0.0024	3.96 × 10^−8^	*CCT6P1*	NCBI gene ID 643253. No summary of function available
*EWAS of gestational weight gain, meta-analysis approach*								
cg16733643	1	41,575,522	Open sea	−0.35	0.065	5.82 × 10^−8^	*SCMH1*	NCBI gene ID 22955. Encodes a protein that is predicted to impact chromatin and histone binding activity and thereby negatively impact transcription of DNA.
EWAS of body mass index, European ancestry								
cg02786370	4	2,747,928	Open sea	−0.02	0.0031	2.12 × 10^−10^	*TNIP2*	NCBI gene ID 79155. Encodes a protein that inhibits NfkappaB-activation, is involved in MAP/ERK-signaling pathway in some cells, and could be involved in endothelial cell apoptosis.
cg19877790	13	35,009,671	Open sea	−0.01	0.0034	2,09 × 10^−8^	*LINC00457*	NCBI gene ID 100874179. Non protein coding RNA.

*Chr* chromosome, *Pos* position, + From the EWAS catalog.

*Replicated in The Norwegian Mother, Father and Child Cohort Study of Assisted Reproductive Technology (MoBa-START), using body mass index at week 30, *p* < 0.05.

**Genome Reference Consortium Human Build 37.

For the EWAS of GWG using a meta-analysis approach, we identified one CpG site that was significant after Bonferroni correction (Table 2, Fig. 1). The ancestry specific EWAS of GWG in women of European and South Asian ancestry did not return any CpG sites after the Bonferroni correction. We found no evidence of systematic inflation in the EWAS of GWG, in the meta-analysis approach (*λ* = 1.157), in women of European ancestry (*λ* = 1.035), or of South Asian ancestry (*λ* = 0.951).

### Replication

To validate our results, we attempted replication of our findings in MoBa-START. Three of the five BMI-related CpG sites (cg02786370, cg19758958, and cg10472537) were replicated in MoBa-START for BMI in gestational week 30 (Supplementary File 1, Supplementary Table 4).

### Consistency across timepoints

To assess consistency in our findings, we did a look up of the BMI and GWG related CpG sites. In European women, all BMI related CpG sites were nominally associated with pre-pregnancy BMI (*p* < 0.05), and had β-coefficients in the same direction of effect as found in the cross-ancestry meta-analysis (Supplementary File 1, Supplementary Table 8). In the South Asian women, DNA methylation at two of the three BMI related CpG sites showed a robust association with pre-pregnancy BMI (*p* < 0.05), and both were in the same direction of effect (Supplementary File 1, Supplementary Table 8). In the EWAS of GWG in the women of South Asian ancestry, we found nominal significance for only one of the BMI related CpG sites (Supplementary File 1, Supplementary Table 7).

For the GWG related CpG site, we found no robust association with pre-pregnancy BMI or BMI at gestation week 28 ± 2 (Supplementary File 1, Supplementary Tables 6 and 9).

### Association between replicated CpG sites and selected cardiometabolic parameters

We assessed associations between the BMI or GWG related CpG sites and clinically relevant phenotypes. The three BMI related CpG sites were also associated with several parameters related to cardiometabolic health (Supplementary File 1, Tables 10 to 12). HDL-cholesterol was positively associated with cg10472537, while DNA methylation of the three CpGs was negatively associated with BMI, blood pressure, triglycerides, and glucose-related traits (Supplementary File 1, Tables 10 to 12). Parity was not associated with any of the BMI or GWG related CpG sites (Supplementary File 1, Tables 10 to 13). The GWG related CpG site was associated with levels of C-peptide, insulin, HOMA-IR and gestational diabetes mellitus using WHO 2013 criteria (Supplementary File 1, Table 13). The tested CpG sites showed some association with the different cell types (Supplementary File 1, Tables 10 to 13). To verify that our results were not caused by limitations to the Houseman method for cell composition, we also performed the EWAS of BMI using an alternative method for cell type estimation, FlowSorted.Blood.EPIC (https://github.com/immunomethylomics/FlowSorted.BloodExtended.EPIC). There were minor differences in our main findings (Supplementary file 1, Supplementary Table 14). One of the three sites identified in the meta-analysis and replicated in MoBa was observed to be close to, but did not quite reach genome-wide significance using the Bonferroni correction (6 × 10−8) (Supplementary file 1, Supplementary Table 14).

### Pathway analyses

To assess potential biologically relevant pathways associated with DNA methylation of BMI or GWG related CpG sites, we checked all sites significant using a cut-off of FDR 5% (Supplementary file 1, Supplementary Tables 1 and 2) for associated gene ontology pathways. None of the sites were significantly (FDR < 5%) associated with any gene ontology pathways.

### Association between the three discovered CpG sites and genotype (mQTL)

To identify related genetic variants, we performed mQTL analysis of the BMI or GWG related CpG sites. DNA methylation of one of the three BMI related CpG sites, cg02786370, was associated with genetic variants (*p*-value < 5 × 10^−8^, Table 3); 231 genetic variants in cis and 81 in trans among the Europeans (Table 3). The identified genetic variants were in three linkage disequilibrium blocks (R^2^ ranging from 1.0 to 0.29). After pruning the results, one genetic variant remained from each block for lookups in the Phenoscanner. Phenotypes related to these genetic variants with nominal significance (*p* < 10^−5^) are displayed in Table 3. rs10119911, associated with cg02786370 in trans, was nominally significant in summary data from a previous GWAS of BMI [43, 44]. rs9472010 associated with cg02786370 in trans was nominally significant in summary data from a previous GWAS of coronary artery disease [45]. For the GWG related CpG site, cg16733643, we identified 418 mQTLs in cis, and we were left with eleven variants from eleven linkage disequilibrium blocks (R^2^ ranging from 1.0 to 0.15) after pruning, none of the gene variants were previously associated with any phenotype.

**Table 3:** Top methylation quantitative trait loci significantly associated with CpG sites from epigenome wide association study of BMI or GWG in gestational week 28 ± 2, and their associated phenotypes in published genome wide association studies (*p* < 0.001).

CpG	Ancestry	mQTLs	Number of variants	Top gene variant	Chromosome	Associated phenotypes	Kolonne2	Kolonne3
						Phenotype	Direction	p-value
cg02786370	European	Cis	231	rs7694454	4	Basophile count (PMID: 27863252)	−	4.33e-04
		Trans	13	rs9472010	6	Coronary artery disease (PMID: 29212778)	+	7.10e-04
			68	rs10119911	9	BMI (PMID: 25673413, 26426971).	−	5.16e-04
						Hemoglobin concentration (PMID: 27863252)	+	7.21e-04
						Allergic disease (PMID: 29083406)	−	2.39e-05
						Asthma (PMID: 29273806)	−	5.98e-04
cg164733643	European	Cis	418	rs12059241	1			

*EWAS* epigenome wide association study, *GWAS* genome wide association study, *mQTL* methylation quantitative trait loci,

*MoBa-START* the Norwegian mother, father and child cohort study of assisted reproductive technology.

*using body mass index at week 30, *p* < 0.05.

### Lookups in databases

To assess previous associations between the BMI and GWG related CpG sites, we searched for phenotypes in EWAS catalog, EWAS atlas, GoDMC database and Phenoscanner. In the EWAS atlas, smoking has previously been identified in association with hypomethylation of cg02786370 (PMID: 33593402) and cg19758958 (PMID: 33593402 and 33823916). Hypermethylation of cg16733643 is previously associated with preterm birth (PMID: 28428831) and Down syndrome (PMID: 29601581), while hypomethylation is previously associated with estrogen exposure (PMID: 31039828). We found no hits for any of the replicated CpG sites in EWAS catalog, GoDMC database, or Phenoscanner.

## Discussion

To the best of our knowledge, this is the first EWAS of BMI and GWG during pregnancy. In a cross-ancestry meta-analysis, we identified five CpG sites whose DNA methylation levels were associated with BMI at the beginning of the third trimester of pregnancy after Bonferroni correction, three of which were replicated in an independent cohort (comprising mainly European participants). We also identified one CpG site significant after Bonferroni correction in the EWAS of GWG. However, we were not able to attempt replication of this site in MoBa-START due to different cohort designs (DNA methylation quantified before or after the GWG). Two of the three BMI related CpG sites were robustly associated with BMI across different time points before and during pregnancy in samples from women of both South Asian and European ancestry. All three BMI related sites were negatively associated with blood pressure, glucose-related traits, and triglycerides, and positively with HDL-cholesterol levels. cg02786370 was associated with rs10119911 and rs9472010 which were nominally significant in association with BMI [43, 44] and coronary artery disease [45], respectively, in look-ups of GWAS summary data. The GWG related CpG site showed association with glucose related parameters.

Two of the three BMI related CpG sites were located in annotated genes with a NCBI summary of function. The CpG site, cg02786370, is located in the ‘TNFAIP3 Interacting Protein 2’ (*TNIP2*) gene [46]. Its expression inhibits the activation of Nuclear Factor-kappa-B, a transcription factor involved in inflammation and apoptosis [46]. The GWAS Catalog [47] shows that this gene was associated with systolic blood pressure in a study of 750,000 individuals [48]. In our analysis, cg02786379 was negatively associated with systolic blood pressure. Despite finding an association, we cannot conclude on causation. Also, the location of the CpG sites in the open sea region makes it difficult to hypothesize on potential direction of effect on gene expression. The CpG site cg19758958 is located in the promoter region of the gene AHNAK nucleoprotein (*AHNAK*), which encodes a structural scaffold protein that may be involved in cell structure and migration, blood-brain barrier, tumor metastasis, and cardiac calcium channels [49]. Interestingly, data from the GWAS Catalog [47] shows that this gene has been associated with BMI [50] and HDL-cholesterol [51] in a study using data from the United Kingdom biobank. However, we did not find a significant association between DNA methylation levels of cg19758958 and HDL-cholesterol in our analyses. Our search in the EWAS atlas revealed some previous associations between smoking and hypomethylation of cg0278360 and cg19758958. Despite our efforts to control for smoking in our analyses, we cannot rule out that the results are somehow influenced by smoking.

A bidirectional Mendelian Randomization study suggested that most BMI-associated CpG sites were induced by being overweight, and not that DNA methylation levels impact BMI [11]. Our identified mQTLs may indicate the opposite, although our cross-sectional design does not allow us to conclude on causality. We found several genetic variants associated with DNA methylation at the replicated cg02786379 in both cis and trans in Europeans, as well as for cg16733643 suggesting a genetic component influencing differential methylation, that in turn may influence BMI and/or GWG, and further studies should explore how mQTLs and DNA methylation may affect complex traits. Interestingly, genetic variants related to cg02786379 were nominally associated with BMI [43, 44] and coronary artery disease [45], in GWAS summary data, although they did not reach genome-wide significance. Hence, EWAS may help identify disease-related genetic variants which may be important to understanding disease mechanisms and to develop potential candidates for prevention or treatment. The datasets used for the GWAS of BMI and coronary artery disease consist largely of individuals of European ancestry [43, 45]. There are differences in allele frequencies, linkage disequilibrium, and differentially methylated CpG sites across ancestry [43, 52]. To address the systematic difference between women of European and South Asian ancestry and retain the statistical power, we combined and performed a meta-analysis of the EWAS of BMI in European and South Asian women. Despite that none of the CpG sites was statistically significant in the EWAS of BMI in Europeans and South Asians separately, all but one of the effect sizes were in the same direction of effect across ancestry. Our study indicates that a cross-ancestry approach may potentiate the possibility of identifying true positive variants that are replicated in independent cohorts.

We found no overlap between the 42 CpG sites associated with BMI at the FDR < 5% and the top 76 CpG sites identified in a meta-analysis of 18 studies of BMI and/or waist circumference in non-pregnant populations [10]. The lack of overlap could be due to epigenetic differences in men and women, as a twin study has suggested sex-specific genetic factors that influence variation in BMI [53]. In this context, it can be hypothesized that the differentially methylated CpG sites are linked to mechanisms specific to pregnancy. However, as many of the CpG sites were also associated with pre-pregnancy BMI, combined with the lack of CpG sites associated with GWG, the identified CpG sites could be specific to BMI in females of reproductive age, and not specific to pregnancy.

The strengths of this study are the high participation rate which minimizes the risk of selection bias as well as a wide distribution of BMI levels. In addition, several of the CPG sites were consistently associated with BMI at different time points. The phenotypically well-characterized sample enabled exploration of associations with cardiometabolic factors, and the use of measured height and weight for calculation of BMI gives more accurate values than self-reported BMI. Since there is a strong and well-known clinical association between BMI and cardiometabolic parameters, our association analyses of methylated CpG sites and association to cardiometabolic parameters are prone to confounding. Our results can only indicate a multifactorial and complex interplay between biological mechanisms and clinical outcomes related to body composition. An important limitation of our study is that gained fat mass and fluid retention may vary largely among pregnant women [54], and BMI in week 28 may capture different weight gain mechanisms in different women. GWG is a combination of the placenta, fetus, and amniotic fluid, as well as hypertrophy and hyperplasia of maternal tissues [55]. However, during the first and second trimester, most of the GWG is due to maternal components, where growth of the uterus and breasts and expansion of blood volume account for a larger proportion than fat accumulation [55]. Variations in fat mass and fluid retention may have been especially challenging for GWG, and our one significant CpG site has not been validated in an independent cohort. The lack of significant results in the EWAS of GWG could be due to both inter- and intraindividual differences in tissue composition. Further, our sample has limited statistical power and can only detect CpG sites with high effect sizes and low variation [12]. We cannot rule out that the two sites that did not replicate in MoBa-START are due to different timing of sampling (week 28 in EPIPREG vs week 18 in MoBa-START), however, this is not likely due to the robustness across BMI timepoints in our sample. In addition, it is important to note that we cannot by using estimates and not actual measurement of cell composition rule out that the DNA methylation sites we discover here are driven by differences in cell composition. As of now, there is no consensus in the epigenetic research field on preferred method for correction. Our results persisted after adjustment for a different estimation method for cell composition, suggesting that our findings are robust despite limitations to the Houseman method.

## Conclusion

We identified one CpG site associated with GWG from pre-pregnancy to gestational week 28, and five CpG sites associated with BMI in gestational week 28 after Bonferroni correction, where three were replicated in an independent cohort. cg02786370 is located in the gene *TNFAIP3*, which has previously been associated with higher blood pressure. The CpG site cg19758958 is located in the promoter region of the gene *AHNAK* that was associated with HDL cholesterol and BMI. We identified associated mQTLs to cg02786370 which was associated with BMI and coronary artery disease with nominal significance in GWAS summary data. Methylation at the CpG sites identified was also associated with cardiometabolic parameters in our material such as blood pressure, blood lipids, and blood glucose levels.

### Supplementary information

Supplementary file 1

## Data Availability

Due to strict regulations for genetic data and privacy protection of patients in Norway, all requests for data access are processed by the STORK Groruddalen project’s steering committee. Data access requests can be filed to the primary investigator of STORK Groruddalen (a.m.l.brand@medisin.uio.no) or the primary investigator of EPIPREG (christine.sommer@medisin.uio.no). The data in the MoBa START project used for replication analyses in this study is available from NIPH, but restrictions apply regarding the availability of these data. Access can be obtained by applying to NIPH at https://www.fhi.no/en/studies/moba/. Access can only be given after approval by the Norwegian Ethical committees on the grounds that the applications are consistent with the consent provided.
