# Supplementary material for: Epigenome-wide association study of DNA methylation in maternal blood leukocytes with BMI in pregnancy and gestational weight gain
Source: Int J Obes (Lond). 2024 Jan 13;48(4):584–93. doi: 10.1038/s41366-024-01458-x (PMC10978488; doi:10.1038/s41366-024-01458-x)
Supplement: Supplementary file 1 [file 41366_2024_1458_MOESM1_ESM.docx]

Supplementary file 1

Innholdsfortegnelse

[Supplementary information about replication in The Norwegian Mother, Father and Child Study of Assisted Reproductive Technology (MoBa-START) 3](#_Toc147223010)

[Supplementary table 1- Significant CpG sites (false discovery rate 5%) from the epigenome wide association study of body mass index in gestational week 28±2 and DNA methylation in peripheral white blood cells in women of European ancestry 4](#_Toc147223011)

[Supplementary table 2- Significant CpG sites (false discovery rate 5%) from the epigenome wide association study of body mass index in gestational week 28±2 and DNA methylation in peripheral white blood cells using a meta-analysis approach 5](#_Toc147223012)

[Supplementary table 3- Significant CpG sites (false discovery rate 5%) from the epigenome wide association study of gestational weight gain (pre-pregnant weight to weight in gestational week 28±2) using a meta-analysis approach 8](#_Toc147223013)

[Supplementary table 4- Replication of the identified CpG sites (false discovery rate 5%) from the epigenome wide association study of body mass index in gestational week 28±2 and DNA methylation in peripheral white blood cells using a meta-analysis approach in The Norwegian Mother, Father and Child Study of Assisted Reproductive Technology (MoBa-START) epigenome wide association study of body mass index in gestational week 30 and DNA methylation in peripheral white blood cells 8](#_Toc147223014)

[Supplementary table 5- Look up of identified CpG sites (false discovery rate 5%) from the epigenome wide association study of body mass index in gestational week 28±2 and DNA methylation in peripheral white blood cells using a meta-analysis approach, in the separate epigenome wide association studies of women of European and South Asian ancestry 11](#_Toc147223015)

[Supplementary table 6- Look up of identified CpG sites (false discovery rate 5%) from the epigenome wide association study of gestational weight gain (pre-pregnant weight to weight in gestational week 28±2) and DNA methylation in peripheral white blood cells using a meta-analysis approach, in the separate epigenome wide association studies of women of European and South Asian ancestry 13](#_Toc147223016)

[Supplementary table 7- Look up of identified CpG sites (false discovery rate 5%) from the epigenome wide association study of body mass index in gestational week 28±2 and DNA methylation in peripheral white blood cells using a meta-analysis approach, in the separate epigenome wide association studies of gestational weight gain (pre-pregnant weight to weight in gestational week 28±2) in women of European and South Asian ancestry 13](#_Toc147223017)

[Supplementary table 8- Look up of identified CpG sites (false discovery rate 5%) from the epigenome wide association study of body mass index in gestational week 28±2 and DNA methylation in peripheral white blood cells using a meta-analysis approach, in the separate epigenome wide association studies of pre-pregnant body mass index and DNA in women of European and South Asian ancestry 16](#_Toc147223018)

[Supplementary table 9- Look up of identified CpG sites (false discovery rate 5%) from the epigenome wide association study of gestational weight gain (pre-pregnant weight to weight in gestational week 28±2) and DNA methylation in peripheral white blood cells using a meta-analysis approach, in the separate epigenome wide association studies of pre-pregnant body mass index and DNA in women of European and South Asian ancestry 18](#_Toc147223019)

[Supplementary table 10- Analysis of cardiometabolic parameters in association with DNA methylation of cg02786370 in linear regression models adjusted for age, smoking and estimated cell composition. 18](#_Toc147223020)

[Supplementary table 11- Analysis of cardiometabolic parameters in association with DNA methylation of cg19758958 in linear regression models adjusted for age, smoking and estimated cell composition 20](#_Toc147223021)

[Supplementary table 12- Analysis of cardiometabolic parameters in association with DNA methylation of cg10472537 in linear regression models adjusted for age, smoking and estimated cell composition 22](#_Toc147223022)

[Supplementary table 13- Analysis of cardiometabolic parameters in association with DNA methylation of cg16733643in linear regression models adjusted for age, smoking and estimated cell composition 24](#_Toc147223023)

[Supplementary table 14- Comparison of Houseman and FlowSorted.Blood.EPIC methods for cell type estimation in epigenome wide association study of body mass index in gestational week 28±2 and DNA methylation in peripheral white blood cells using a meta-analysis approach 26](#_Toc147223024)

[Supplementary figure 1- Flow chart of the study 28](#_Toc147223025)

# Supplementary information about replication in The Norwegian Mother, Father and Child Study of Assisted Reproductive Technology (MoBa-START)

The Norwegian Mother, Father and Child Cohort study (MoBa) participants filled out a series of questionnaires during pregnancy and at multiple time points after delivery. The current study is based on version V12 of the quality-assured data files released for research in 2019. This study was approved by the Regional Committees for Medical and Health Research Ethics of South East Norway (#2017/1362). All participants in MoBa have provided written informed consent. The establishment of MoBa and initial data collection was based on a license from the Norwegian Data Protection Agency and an approval from the Regional Committees for Medical and Health Research Ethics. The MoBa cohort is now regulated by the Norwegian Health Registry Act.

# Supplementary table 1- Significant CpG sites (false discovery rate 5%) from the epigenome wide association study of body mass index in gestational week 28±2 and DNA methylation in peripheral white blood cells in women of European ancestry

| CpG-site | Relation to Island | Position | Chromosome | UCSC reference gene name | Gene function | β | SE | P-value |
| --- | --- | --- | --- | --- | --- | --- | --- | --- |
| cg02786370* | OpenSea | 2747928 | chr4 | *TNIP2* | NCBI gene ID 79155. Encodes a protein that inhibits NFkappaB-activation, is involved in MAP/ERK-signaling pathway in some cells, and could be involved in endothelial cell apoptosis. | -0,0229 | 0,0035 | 2.363e-10 |
| cg19877790* | OpenSea | 35009671 | Chr13 | *LINC00457* | NCBI gene ID 100874179. Non protein coding RNA. | -0,0120 | 0,0034 | 2,085e-08 |

UCSC: University of California, Santa Cruz; BMI: body mass index; GW: gestational week; β: beta-coefficient; SE: standard error; NCBI: National center for biotechnology information. *Significant using Bonferroni cut-off of *p*<0.05/806 236= 6*10-8

# Supplementary table 2- Significant CpG sites (false discovery rate 5%) from the epigenome wide association study of body mass index in gestational week 28±2 and DNA methylation in peripheral white blood cells using a meta-analysis approach

| CpG-site | Relation to Island | Position | Chromosome | UCSC reference gene name | Gene function | β | SE | P-value |
| --- | --- | --- | --- | --- | --- | --- | --- | --- |
| cg02786370* | OpenSea | 2747928 | chr4 | *TNIP2* | NCBI gene ID 79155. Encodes a protein that inhibits NFkappaB-activation, is involved in MAP/ERK-signaling pathway in some cells, and could be involved in endothelial cell apoptosis. | -0,0198 | 0,0031 | 2.124e-10 |
| cg19758958* | OpenSea | 62319222 | chr11 | *Promoter region of AHNAC* | NCBI gene ID 79026. Encodes a structural scaffold protein that may play a role in blood-brain barrier formation, cell structure and migration, cardiac calcium channel regulation, and tumor metastasis | -0,0107 | 0,0018 | 1.116e-09 |
| cg10472537* | OpenSea | 105348800 | chr2 |  |  | -0,0111 | 0,002 | 2.05e-08 |
| cg16444328* | OpenSea | 171279429 | chr5 |  |  | -0,0082 | 0,0015 | 3.763e-08 |
| cg24911837* | N_Shore | 65227864 | chr7 | *CCT6P1* | NCBI gene ID 643253. No summary of function available | -0,0131 | 0,0024 | 3.963e-08 |
| cg23191724 | OpenSea | 70267556 | chr11 | *CTTN* | NCBI gene ID 2017. Encodes a protein regulating cell contacts and organisation of cytoskeleton and cell adhesion of epithelium and cancer cells. Overexpressed in squamous cell carcinomas of head and neck, as well as breast cancer, where it contributes to tumour cell invasion and metastasis. | -0,0178 | 0,0033 | 8.47e-08 |
| cg01270753 | OpenSea | 101944336 | chr9 |  |  | -0,017 | 0,0032 | 1.008e-07 |
| cg26050822 | OpenSea | 30320193 | chrX |  |  | -0,0241 | 0,0046 | 1.249e-07 |
| cg08298765 | OpenSea | 123983480 | chr9 |  |  | -0,0072 | 0,0014 | 1.948e-07 |
| cg20887442 | OpenSea | 71725424 | chr11 | *NUMA1* | NCBI gene ID 4926. Encodes a protein involved in formation and organization of the mitotic spindle during cell division. | -0,0082 | 0,0016 | 2.533e-07 |
| cg05551937 | S_Shore | 57776726 | chr4 | *REST* | NCBI gene ID 5978. Encodes a protein that can act as an oncogene or tumor supressor, depending on the cellular context. Can repress gene transcription by binding a silencer element, can possibly be a negative regulator of neurogenesis. | 0,0119 | 0,0023 | 2.899e-07 |
| cg02702424 | OpenSea | 30654871 | chr3 | *TGFBR2* | NCBI gene ID 7048. Encodes a protein that forms a heterodimeric complex with TGF-beta receptor type-1. Involved in TGF-signalling that regulates a variety of cellular functions. | -0,0093 | 0,0018 | 3.516e-07 |
| cg03340148 | OpenSea | 11971431 | chr16 | *GSPT1* | NCBI gene ID 2935. No summary of function available. | -0,0093 | 0,0018 | 3.67e-07 |
| cg12737110 | Island | 219861126 | chr2 |  |  | -0,0132 | 0,0026 | 3.944e-07 |
| cg06222414 | N_Shelf | 44636919 | chr20 | *MMP9* | NCBI gene ID 4318. Encodes a matrix metalloproteinase which is envolved in breakdown of extracellular matrix (this protein targets type IV and V collagen) in both normal physiological processes and diseases. | -0,015 | 0,003 | 5.26e-07 |
| cg10251538 | OpenSea | 108800886 | chr3 | *MORC1* | NCBI gene ID 27136. Encodes a protein that in mouse has been suggested to be involved in spermatogenesis. | -0,0133 | 0,0027 | 6.148e-07 |
| cg18972123 | OpenSea | 54532059 | chr10 | *MBL2* | NCBI gene ID 4153. Encodes a protein involved in the innate immune system, which recognizes and binds to mannose and N-acetylglycosamine on microorganisms (PAMPs). | -0,0092 | 0,0018 | 6.59e-07 |
| cg20715780 | OpenSea | 186814701 | chr1 | *PLA2G4A* | NCBI gene ID 5321. Encodes a protein in the cytosolic phospholipase A2-family that is involved in the arachidonic acid-metabolism., which is important for inflammatory responses. | -0,0079 | 0,0016 | 6.637e-07 |
| cg22851864 | OpenSea | 5688470 | chr10 | *ASB13* | NCBI gene ID 79754. Encodes a protein involed to couple supressor of cytokine signalling proteins and elongin binding partners, possibly for their degradation. | -0,0326 | 0,0066 | 6.661e-07 |
| cg27179379 | OpenSea | 63988325 | chr1 | *EFCAB7* | NCBI gene ID 84455. No summary of function available | -0,0293 | 0,0059 | 7.065e-07 |
| cg11254085 | Island | 109744679 | chr2 | *SH3RF3* | NCBI gene ID 344558. No summary of function available | 0,0107 | 0,0022 | 7.112e-07 |
| cg22528063 | OpenSea | 127114914 | chr9 | *LOC100129034* | NCBI gene ID 10012903. No summary of function available. | -0,0066 | 0,0013 | 7.425e-07 |
| cg12404216 | S_Shore | 46743199 | chr3 | *TMIE* | NCBI gene ID 259236. Encodes an inner ear transmembrane protein that could be involved in maturation of cochlear hair cells. Among other genes responsible for recessive non-syndomic deafness. | -0,008 | 0,0016 | 8.929e-07 |
| cg15239145 | S_Shore | 128285392 | chr2 | *IWS1* | NCBI gene ID 55677. No summary of function available. | -0,0101 | 0,0021 | 9.059e-07 |
| cg06088069 | S_Shore | 75895604 | chr14 | *JDP2* | NCBI gene ID 122953. No summary of function available. | -0,0063 | 0,0013 | 9.366e-07 |
| cg02693156 | OpenSea | 9903454 | chr12 |  |  | -0,0477 | 0,0098 | 1.072e-06 |
| cg12404181 | OpenSea | 5688515 | chr10 | *ASB13* | NCBI gene ID 79754. Encodes a protein involed to couple supressor of cytokine signalling proteins and elongin binding partners, possibly for their degradation. | -0,0279 | 0,0057 | 1.133e-06 |
| cg27560175 | OpenSea | 48118501 | chr20 |  |  | -0,02 | 0,0041 | 1.179e-06 |
| cg05271333 | OpenSea | 43084407 | chr6 | *PTK7* | NCBI gene ID 5754. Encodes a protein that is a member of the receptor protein tyrosine kinase family. Involved in Wnt signaling and other cellular processes. | -0,0118 | 0,0024 | 1.208e-06 |
| cg13823169 | N_Shelf | 139776893 | chr9 |  |  | -0,0085 | 0,0018 | 1.369e-06 |
| cg20786944 | OpenSea | 29606019 | chr21 |  |  | -0,0077 | 0,0016 | 1.426e-06 |
| cg02543993 | OpenSea | 5736195 | chr7 | *RNF216* | NCBI gene ID 54476. Encodes a protein that interacts with serine/threonine protein kinase, receptor-interacting protein that is involved in inhibition of TNF- and IL1-induced NF-kappa B activation pathways. May also be involved in ubiquitination of substrates. | -0,0201 | 0,0042 | 1.635e-06 |
| cg15198407 | OpenSea | 80841737 | chr10 | *ZMIZ1* | NCBI gene ID 57178. Encodes a protein that is member of the protein inhibitor of activated STAT-proteins. Regulates the activity of various transcription factors, and could play arole in sumoylation. | -0,0084 | 0,0018 | 1.734e-06 |
| cg05630316 | OpenSea | 56830679 | chr8 | *LYN* | NCBI gene ID 4067. Encodes a tyrosine protein kinase. May be involved in regulation of mast cells and erythroid differentiatin. | -0,0084 | 0,0018 | 1.77e-06 |
| cg01885635 | N_Shore | 40566085 | chr3 | *ZNF621* | NCBI gene ID 285268. No summary of function available. | -0,0079 | 0,0016 | 1.828e-06 |
| cg23223006 | S_Shore | 44203109 | chrX | *EFHC2* | NCBI gene ID 8258. Encodes a protein that could be involved in development of epilepsy and has been associated to fear recognition in Turner syndrome. | -0,0174 | 0,0037 | 2.073e-06 |
| cg06988896 | S_Shelf | 78426368 | chr15 |  |  | -0,0079 | 0,0017 | 2.162e-06 |
| cg25691233 | OpenSea | 5721828 | chr7 | *RNF216* | NCBI gene ID 54476. Encodes a protein that interacts with serine/threonine protein kinase, receptor-interacting protein that is involved in inhibition of TNF- and IL1-induced NF-kappa B activation pathways. May also be involved in ubiquitination of subst | 0,0107 | 0,0023 | 2.198e-06 |
| cg25364972 | OpenSea | 217075573 | chr2 |  |  | -0,0103 | 0,0022 | 2.21e-06 |

UCSC: University of California, Santa Cruz; BMI: body mass index; GW: gestational week; β: beta-coefficient; SE: standard error; NCBI: National center for biotechnology information. *Significant using Bonferroni cut-off of *p*<0.05/806 236= 6*10-8

# Supplementary table 3- Significant CpG sites (false discovery rate 5%) from the epigenome wide association study of gestational weight gain (pre-pregnant weight to weight in gestational week 28±2) using a meta-analysis approach

| CpG-site | Relation to Island | Position | Chromosome | UCSC reference gene name | Gene function | β | SE | P-value |
| --- | --- | --- | --- | --- | --- | --- | --- | --- |
| cg16733643* | OpenSea | 41575522 | Chr1 | *SCMH1* | NCBI gene ID 22955. Encodes a protein that is predicted to impact chromatin and histone binding activity and thereby negatively impact transcription of DNA. | -0,354 | 0,065 | 5,852e-08 |

UCSC: University of California, Santa Cruz; BMI: body mass index; GW: gestational week; β: beta-coefficient; SE: standard error; NCBI: National center for biotechnology information. *Significant using Bonferroni cut-off of *p*<0.05/806 236= 6*10-8

# Supplementary table 4- Replication of the identified CpG sites (false discovery rate 5%) from the epigenome wide association study of body mass index in gestational week 28±2 and DNA methylation in peripheral white blood cells using a meta-analysis approach in The Norwegian Mother, Father and Child Study of Assisted Reproductive Technology (MoBa-START) epigenome wide association study of body mass index in gestational week 30 and DNA methylation in peripheral white blood cells

| CpG-site | β | SE | P-value |
| --- | --- | --- | --- |
| cg02786370* | -0,00194463 | 0,000444544 | 1,38E-05 |
| cg19758958* | -0,001623426 | 0,000339559 | 2,08E-06 |
| cg10472537* | -0,001210037 | 0,000397408 | 0,002404302 |
| cg16444328 | -0,000391267 | 0,000279257 | 0,161570068 |
| cg24911837 |  |  |  |
| cg23191724* | -0,000287498 | 0,000140025 | 0,040377748 |
| cg01270753* | -0,003043285 | 0,000546241 | 3,45E-08 |
| cg26050822 |  |  |  |
| cg08298765 | -0,000224287 | 0,000256285 | 0,381755747 |
| cg20887442 | -0,000206078 | 0,000216425 | 0,341287653 |
| cg05551937 | -3,54E-06 | 7,93E-05 | 0,964351044 |
| cg02702424* | -0,000812649 | 0,000254452 | 0,001459355 |
| cg03340148 | -0,000468906 | 0,00036846 | 0,203525186 |
| cg12737110 | 0,000303935 | 0,000360402 | 0,399299497 |
| cg06222414 | -0,000478159 | 0,000244659 | 0,051002492 |
| cg10251538* | -0,001220425 | 0,000299644 | 5,10E-05 |
| cg18972123 | -0,000416417 | 0,000415502 | 0,316548277 |
| cg20715780 |  |  |  |
| cg22851864* | -0,001924374 | 0,00078729 | 0,014727661 |
| cg27179379 | 4,06E-05 | 3,37E-05 | 0,228216891 |
| cg11254085 |  |  |  |
| cg22528063 | 2,55E-05 | 0,000279156 | 0,927265325 |
| cg12404216 | 0,000254481 | 0,000250829 | 0,310622197 |
| cg15239145 | -6,57E-05 | 6,56E-05 | 0,316591918 |
| cg06088069* | -0,000713768 | 0,000282171 | 0,011611073 |
| cg02693156 |  |  |  |
| cg12404181 | -0,000786671 | 0,000811739 | 0,33277796 |
| cg27560175 |  |  |  |
| cg05271333 | -0,000252621 | 0,000311494 | 0,417608471 |
| cg13823169* | -0,000709064 | 0,000324217 | 0,029030263 |
| cg20786944 | -0,000488815 | 0,000316833 | 0,123269039 |
| cg02543993* | -0,001580915 | 0,00043636 | 0,000309583 |
| cg15198407 | -0,000211679 | 0,00028271 | 0,454227941 |
| cg05630316* | -0,000812496 | 0,000327501 | 0,013309273 |
| cg01885635 | -0,000443709 | 0,000253501 | 0,080444967 |
| cg23223006 |  |  |  |
| cg06988896* | -0,000809953 | 0,000368451 | 0,028215217 |
| cg25691233 | -0,000435592 | 0,000307478 | 0,156969623 |
| cg25364972 | -0,000211301 | 0,000421791 | 0,616535936 |

β: beta-coefficient; SE: standard error. *Significant using a nominal cut-off of *p*<0.05

# Supplementary table 5- Look up of identified CpG sites (false discovery rate 5%) from the epigenome wide association study of body mass index in gestational week 28±2 and DNA methylation in peripheral white blood cells using a meta-analysis approach, in the separate epigenome wide association studies of women of European and South Asian ancestry

|  | European ancestry | | | South Asian ancestry | | |
| --- | --- | --- | --- | --- | --- | --- |
| CpG-site | **β** | **SE** | **P-value** | **β** | **SE** | **P-Value** |
| cg02786370*+ | -0,02288161 | 0,003495419 | 2.36301695100604e-10 | -0,007867 | 0,006829 | 5.529415e-03 |
| cg19758958*+ | -0,01069584 | 0,002023003 | 2.74388674908523e-07 | -0,01063 | 0,0035 | 3.072576e-03 |
| cg10472537*+ | -0,01 | 0,0023 | 3.230421e-06 | -0,01185 | 0,00398 | 3.461306e-93 |
| cg16444328*+ | -0,0078 | 0,0017 | 8.07759e-06 | -0,09189 | 0,00299 | 2.892889e-03 |
| cg24911837*+ | -0,013 | 0,0028 | 5.339179e-06 | -0,01326 | 0,00449 | 3.607227e-03 |
| cg23191724*+ | -0,01993692 | 0,003854265 | 3.92990551613323e-07 | -0,01159 | 0,006525476 | 7.610609e-02 |
| cg01270753*+ | -0,01865176 | 0,007181888 | 8.4723903023067e-07 | -0,01238 | 0,006241 | 4.764733e-02 |
| cg26050822*+ | -0,022 | 0,0056 | 0.0001262052 | -0,02871 | 0,007809 | 2.919321e-04 |
| cg08298765*+ | -0,00782049 | 0,001612362 | 2.55621162777302e-06 | -0,00543 | 0,00266 | 4.737323e-02 |
| cg20887442* | -0,01012277 | 0,001886281 | 1.93131211236838e-07 | -0,003447 | 0,00301 | 2.591204e-01 |
| cg05551937*+ | 0,012 | 0,0027 | 1.130298e-05 | 0,00252 | 0,00458 | 1.253913e-02 |
| cg02702424* | -0,01092504 | 0,002147575 | 7.06031163930349e-07 | -0,00507 | 0,00351 | 1.528432e-01 |
| cg03340148* | -0,01004 | 0,0021 | 3.04197e-06 | -0,00697 | 0,00371 | 6.264230e-02 |
| cg12737110*+ | -0,0136 | 0,003 | 9.959951e-06 | -0,0118 | 0,005179 | 2.357655e-02 |
| cg06222414*+ | -0,01831912 | 0,003495419 | 3.23892156799444e-07 | -0,00627138 | 0,00569 | 2.357655e-02 |
| cg10251538* | -0,01446 | 0,0029 | 2.027043e-06 | -0,00868 | 0,00586 | 1.382465e-01 |
| cg18972123* | -0,00986 | 0,00219 | 1.086163e-05 | -0,007515 | 0,00339 | 2.934950e-01 |
| cg20715780* | -0,00927 | 0,001889 | 1.765486e-06 | -0,00458 | 0,00294 | 1.260345e-01 |
| cg22851864* | -0,03697 | 0,00763 | 1.836986e-06 | -0,020333 | 0,0128 | 1.112759e-01 |
| cg27179379* | -0,03551314 | 0,006824915 | 3.19014033564102e-07 | -0,0107 | 0,01188 | 3.65870e-01 |
| cg11254085*+ | 0,0108 | 0,0023 | 4.6257e-06 | 0,009739 | 0,005896 | 9.883548e-02 |
| cg22528063*+ | -0,00623 | 0,001568 | 1.068533e-04 | -0,00734 | 0,00247 | 4.220019e-03 |
| cg12404216*+ | -0,00627 | 0,00204 | 2.88306e-03 | -0,0112 | 0,00274 | 8.830329e-05 |
| cg15239145*+ | -0,009617 | 0,00256 | 2.088394e-04 | -0,011 | 0,00345 | 1.920952e-03 |
| cg06088069* | -0,006798 | 0,001492 | 1.016586e-05 | -0,00486 | 0,0025 | 5.987986e-02 |
| cg02693156* | -0,05166 | 0,01094 | 3.203711e-06 | -0,032 | 0,0218 | 1.389046e-01 |
| cg12404181* | -0,03322498 | 0,006603607 | 7.4616328766695e-07 | -0,0116 | 0,0116 | 3.154677e-01 |
| cg27560175* | -0,0197 | 0,0043789 | 9.322211e-06 | -0,02199 | 0,0118 | 6.236356e-02 |
| cg05271333* | -0,01357 | 0,00289 | 3.999791e-06 | -0,007556 | 0,00455 | 9.834458e-02 |
| cg13823169*+ | -0,009797 | 0,002118 | 6.059104e-06 | -0,00311 | 0,00318 | 0.197771e-02 |
| cg20786944* | -0,00906 | 0,001819 | 1.273477e-06 | -0,02313 | 0,00336 | 3.604220e-01 |
| cg02543993*+ | -0,01918 | 0,004776 | 6.992782e-05 | -0,001173 | 0,00874 | 8.386053e-03 |
| cg15198407* | -0,00864821 | 0,001789 | 2.564035e-06 | -0,00766 | 0,01028 | 9.084605e-01 |
| cg05630316*+ | -0,008627 | 0,002037796 | 3.335787e-05 | -0,0104 | 0,0344 | 2.835612e-02 |
| cg01885635*+ | -0,00653 | 0,00204 | 0.001610505 | -0,00766 | 0,00279 | 3.581737e-04 |
| cg23223006*+ | -0,020433 | 0,004155 | 1.338416e-06 | -0,00672 | 0,00279 | 3.889852e-02 |
| cg06988896*+ | -0,00815 | 0,001914 | 3.092968e-05 | -0,00721 | 0,00784 | 3.944910e-02 |
| cg25691233*+ | 0,00897 | 0,00266 | 0.0008342279 | 0,015 | 0,00345 | 5.501463e-04 |
| cg25364972*+ | -0,0102 | 0,00251 | 6.063911e-05 | -0,0104 | 0,00432 | 1.705773e-02 |

BMI: body mass index; GW: gestational week; β: beta-coefficient; SE: standard error. *Significant in the Europeans using a nominal cut-off of *p*<0.05. +Significant in the South Asians using a nominal cut-off of *p*<0.05.

# Supplementary table 6- Look up of identified CpG sites (false discovery rate 5%) from the epigenome wide association study of gestational weight gain (pre-pregnant weight to weight in gestational week 28±2) and DNA methylation in peripheral white blood cells using a meta-analysis approach, in the separate epigenome wide association studies of women of European and South Asian ancestry

|  | European ancestry | | | South Asian ancestry | | |
| --- | --- | --- | --- | --- | --- | --- |
| CpG-site | **β** | **SE** | **p-value** | **β** | **SE** | **P-Value** |
| cg16733643*+ | -0.3601284 | 0.0837631 | 2.248e-05 | -03439215 | 0,103969 | 0,00113487 |

β: beta-coefficient; SE: standard error. *Significant in the Europeans using a nominal cut-off of *p*<0.05. +Significant in the South Asians using a nominal cut-off of *p*<0.05.

# Supplementary table 7- Look up of identified CpG sites (false discovery rate 5%) from the epigenome wide association study of body mass index in gestational week 28±2 and DNA methylation in peripheral white blood cells using a meta-analysis approach, in the separate epigenome wide association studies of gestational weight gain (pre-pregnant weight to weight in gestational week 28±2) in women of European and South Asian ancestry

|  | European ancestry | | | South Asian ancestry | | |
| --- | --- | --- | --- | --- | --- | --- |
| CpG-site | **β** | **SE** | **p-value** | **β** | **SE** | **P-Value** |
| cg02786370 | -0,08939844 | 0,08743927 | 0,306201 | -0,2274882 | 0,1336605 | 0,08858948 |
| cg19758958+ | -0,03526748 | 0,05061932 | 0,4886678 | -0,1576396 | 0,06656563 | 0,02017905 |
| cg10472537 | -0,1115942 | 0,05743537 | 0,05355726 | 0,06196861 | 0,0787362 | 0,4336438 |
| cg16444328 | -0,01566562 | 0,04254879 | 0,7156049 | 0,064874 | 0,05472787 | 0,2461548 |
| cg24911837 | -0,005745803 | 0,07071061 | 0,9352565 | -0,1553963 | 0,08751071 | 0,0777508 |
| cg23191724 | 0,03520192 | 0,09689883 | 0,7157686 | -0,1699796 | 0,1305616 | 0,1919031 |
| cg01270753 | -0,139862425 | 0,092263383 | 0,129501799 | -0,141055355 | 0,119070189 | 0,235336185 |
| cg26050822 | -0,1877895 | 0,1417042 | 0,1839839 | -0,064861 | 0,1563918 | 0,6763179 |
| cg08298765 | -0,0260713 | 0,04065937 | 0,5264496 | -0,06300458 | 0,05222258 | 0,2392443 |
| cg20887442 | 0,01141883 | 0,04731345 | 0,810672 | 0,02895207 | 0,06023129 | 0,6357685 |
| cg05551937* | 0,07306287 | 0,06765838 | 0,2810775 | 0,1521648 | 0,09007114 | 0,09294391 |
| cg02702424 | -0,1368784 | 0,0538694 | 0,01187454 | -0,05050302 | 0,06930693 | 0,4703152 |
| cg03340148+ | -0,04160919 | 0,05260354 | 0,4314692 | -0,1555564 | 0,07201229 | 0,03330454 |
| cg12737110* | -0,1569765 | 0,07436895 | 0,03544124 | -0,01334695 | 0,1043244 | 0,8979769 |
| cg06222414*+ | -0,174539 | 0,08766123 | 0,04684353 | -0,2569094 | 0,1129384 | 0,02366262 |
| cg10251538 | -0,02998798 | 0,07484381 | 0,688606 | 0,08780307 | 0,1114332 | 0,4297207 |
| cg18972123 | -0,02137807 | 0,05516041 | 0,6995228 | -0,0977132 | 0,06605672 | 0,144626 |
| cg20715780 | -0,07783707 | 0,04729335 | 0,1031039 | 0,04313656 | 0,05803094 | 0,4649939 |
| cg22851864 | -0,06559814 | 0,1892631 | 0,7276956 | -0,2844993 | 0,2572332 | 0,2652881 |
| cg27179379 | -0,2935382 | 0,1716432 | 0,08660788 | 0,2100132 | 0,2325385 | 0,3628166 |
| cg11254085 | 0,08039855 | 0,05815098 | 0,1687855 | -0,08484274 | 0,117665 | 0,4694882 |
| cg22528063* | -0,04626165 | 0,03906579 | 0,243126 | -0,08489427 | 0,04932695 | 0,09524813 |
| cg12404216+ | -0,06897786 | 0,05095027 | 0,1789012 | -0,1141454 | 0,05342291 | 0,03783003 |
| cg15239145 | -0,09172117 | 0,06441135 | 0,1557782 | 0,03598209 | 0,06899565 | 0,6052668 |
| cg06088069 | -0,0454909 | 0,0377002 | 0,2349619 | -0,0662839 | 0,05019658 | 0,199097 |
| cg02693156* | 0,2088923 | 0,2743784 | 0,4443586 | -0,3903716 | 0,4417015 | 0,3725622 |
| cg12404181 | -0,1782541 | 0,163519 | 0,2740371 | -0,01939781 | 0,2324435 | 0,9328925 |
| cg27560175 | 0,07972679 | 0,1093767 | 0,4648844 | -0,2245835 | 0,2398468 | 0,3454302 |
| cg05271333 | 0,08906915 | 0,07218503 | 0,2178719 | -0,07554557 | 0,09037156 | 0,4040257 |
| cg13823169 | 0,02649027 | 0,05316626 | 0,6200342 | -0,1007518 | 0,06078462 | 0,103717 |
| cg20786944 | -0,05070897 | 0,04580376 | 0,2726916 | -0,03298638 | 0,06563925 | 0,6191384 |
| cg02543993 | -0,09982838 | 0,118003 | 0,3962423 | -0,2321049 | 0,1726821 | 0,1769711 |
| cg15198407 | -0,05358621 | 0,04504848 | 0,2388644 | 0,3643802 | 0,2072025 | 0,07769791 |
| cg05630316* | -0,08575225 | 0,05099186 | 0,09525937 | -0,1279561 | 0,06507527 | 0,05329683 |
| cg01885635 | 0,01305264 | 0,05125621 | 0,8000774 | -0,1080873 | 0,05503324 | 0,05555986 |
| cg23223006 | -0,0539474 | 0,1041935 | 0,6036861 | -0,05977401 | 0,1577294 | 0,7028101 |
| cg06988896 | -0,0139647 | 0,04784047 | 0,7719565 | -0,03822472 | 0,0677061 | 0,5761197 |
| cg25691233*+ | 0,1134425 | 0,0669559 | 0,09131544 | 0,1682369 | 0,08336887 | 0,04560596 |
| cg25364972 | -0,08939844 | 0,08743927 | 0,306201 | -0,2274882 | 0,1336605 | 0,08858948 |

β: beta-coefficient; SE: standard error. *Significant in the Europeans using a nominal cut-off of *p*<0.05. +Significant in the South Asians using a nominal cut-off of *p*<0.05.

# Supplementary table 8- Look up of identified CpG sites (false discovery rate 5%) from the epigenome wide association study of body mass index in gestational week 28±2 and DNA methylation in peripheral white blood cells using a meta-analysis approach, in the separate epigenome wide association studies of pre-pregnant body mass index and DNA in women of European and South Asian ancestry

|  | European ancestry | | | South Asian ancestry | | |
| --- | --- | --- | --- | --- | --- | --- |
| CpG-site | **β** | **SE** | **P-value** | **β** | **SE** | **P-value** |
| cg02786370* | -0,003000837 | 0,000506401 | 8,74E-09 | -0,001358862 | 0,000948844 | 0,154175102 |
| cg19758958*+ | -0,001661267 | 0,000331075 | 9,10E-07 | -0,001666227 | 0,000588402 | 0,00526056 |
| cg10472537*+ | -0,001611373 | 0,000384073 | 3,62E-05 | -0,00205943 | 0,000668591 | 0,002458086 |
| cg16444328*+ | -0,001022874 | 0,000239613 | 2,66E-05 | -0,001538128 | 0,000425369 | 0,000407223 |
| cg24911837*+ | -0,002089322 | 0,000461347 | 8,65E-06 | -0,001781001 | 0,000759983 | 0,020408747 |
| cg23191724* | -0,000861909 | 0,000183128 | 3,90E-06 | -0,000572498 | 0,000338973 | 0,093298947 |
| cg01270753* | -0,00272518 | 0,000572599 | 3,06E-06 | -0,001821707 | 0,000932835 | 0,052683797 |
| cg26050822* | -0,010528323 | 0,010528323 | 1,25E-07 | -0,023977236 | 0,021733148 | 0,266199881 |
| cg08298765* | -0,000958691 | 0,000224275 | 2,60E-05 | -0,000571218 | 0,000383789 | 0,138740062 |
| cg20887442* | -0,001683913 | 0,000315725 | 1,93E-07 | -0,000613435 | 0,000507576 | 0,228722039 |
| cg05551937* | 0,000616215 | 0,000175134 | 0,000503099 | 0,000405117 | 0,0002902 | 0,164765899 |
| cg02702424* | -0,001039174 | 0,000259032 | 7,66E-05 | -0,000683181 | 0,000437848 | 0,120778872 |
| cg03340148* | -0,001636158 | 0,000350778 | 4,72E-06 | -0,000624297 | 0,000611957 | 0,309281651 |
| cg12737110*+ | -0,001507886 | 0,000379657 | 8,99E-05 | -0,001455995 | 0,00067635 | 0,032928812 |
| cg06222414* | -0,001473363 | 0,000336912 | 1,71E-05 | -0,000356868 | 0,000574686 | 0,535549982 |
| cg10251538*+ | -0,0015245 | 0,000330419 | 5,92E-06 | -0,001416863 | 0,000639032 | 0,028103509 |
| cg18972123* | -0,001559696 | 0,000362039 | 2,25E-05 | -0,000923608 | 0,000576679 | 0,111334683 |
| cg20715780* | -0,001524789 | 0,00031458 | 2,04E-06 | -0,00088084 | 0,000492282 | 0,075570876 |
| cg22851864*+ | -0,005623676 | 0,001110042 | 7,21E-07 | -0,003935169 | 0,001816283 | 0,031833342 |
| cg27179379* | -0,000250802 | 7,85E-05 | 0,001555378 | -0,000187318 | 0,00026232 | 0,476278901 |
| cg11254085*+ | 0,000240001 | 5,66E-05 | 2,97E-05 | 0,000325984 | 0,000160612 | 0,044149642 |
| cg22528063*+ | -0,00078102 | 0,00025816 | 0,002704606 | -0,001003711 | 0,000412233 | 0,016065075 |
| cg12404216*+ | -0,000850089 | 0,000269957 | 0,001808155 | -0,001261208 | 0,000379758 | 0,001124539 |
| cg15239145*+ | -0,000468923 | 0,000137545 | 0,00074317 | -0,000540301 | 0,000176583 | 0,002622 |
| cg06088069*+ | -0,001057536 | 0,000252388 | 3,70E-05 | -0,000851303 | 0,000417181 | 0,043031605 |
| cg02693156* | -0,000992425 | 0,000197465 | 8,75E-07 | -0,000497296 | 0,000366233 | 0,176530621 |
| cg12404181*+ | -0,005364278 | 0,001062855 | 7,91E-07 | -0,004281372 | 0,001871688 | 0,023559007 |
| cg27560175* | -0,001775822 | 0,000329581 | 1,47E-07 | -0,002168808 | 0,001327335 | 0,104351068 |
| cg05271333* | -0,001846541 | 0,000385444 | 2,65E-06 | -0,000836785 | 0,000630851 | 0,186696869 |
| cg13823169*+ | -0,001681814 | 0,000348033 | 2,18E-06 | -0,001193104 | 0,000535412 | 0,027332553 |
| cg20786944* | -0,001373912 | 0,000298755 | 6,34E-06 | -0,000415154 | 0,000558793 | 0,458667438 |
| cg02543993*+ | -0,001932304 | 0,000496904 | 0,000124834 | -0,001918508 | 0,000863495 | 0,02778453 |
| cg15198407* | -0,001255871 | 0,000260114 | 2,23E-06 | -0,000203461 | 0,000684775 | 0,766782311 |
| cg05630316*+ | -0,001040221 | 0,000309878 | 0,00089275 | -0,000959353 | 0,000514152 | 0,063995912 |
| cg01885635*+ | -0,000920585 | 0,000271299 | 0,000786134 | -0,001350876 | 0,000393717 | 0,00077561 |
| cg23223006 | NA | NA | NA | NA | NA | NA |
| cg06988896*+ | -0,00140376 | 0,000314703 | 1,17E-05 | -0,001223929 | 0,000576187 | 0,035283363 |
| cg25691233*+ | 0,00089211 | 0,000267467 | 0,00096186 | 0,001225171 | 0,000451228 | 0,007395771 |
| cg25364972*+ | -0,001314515 | 0,000400413 | 0,001152341 | -0,002025159 | 0,000682344 | 0,003487321 |

β: beta-coefficient; SE: standard error. *Significant in the Europeans using a nominal cut-off of *p*<0.05. +Significant in the South Asians using a nominal cut-off of *p*<0.05.

# Supplementary table 9- Look up of identified CpG sites (false discovery rate 5%) from the epigenome wide association study of gestational weight gain (pre-pregnant weight to weight in gestational week 28±2) and DNA methylation in peripheral white blood cells using a meta-analysis approach, in the separate epigenome wide association studies of pre-pregnant body mass index and DNA in women of European and South Asian ancestry

|  | European ancestry | | | South Asian ancestry | | |
| --- | --- | --- | --- | --- | --- | --- |
| CpG-site | **β** | **SE** | **p-value** | **β** | **SE** | **P-Value** |
| cg16733643 | -0.001530853 | 0.003335475 | 0.6458053 | -0.01041436 | 0.005709174 | 0,06867311 |

β: beta-coefficient; SE: standard error

# Supplementary table 10- Analysis of cardiometabolic parameters in association with DNA methylation of cg02786370 in linear regression models adjusted for age, smoking and estimated cell composition.

|  | European ancestry | | | South asian ancestry | | | Meta analysis | | |
| --- | --- | --- | --- | --- | --- | --- | --- | --- | --- |
| cg02786370 | **M-value** | **SE** | **P-value** | **M-value** | **SE** | **P-value** | **M-value** | **SE** | **P-value** |
| Age (whole years) | -0,007720406 | 0,003958053 | 0,052031538 | 0,003195069 | 0,00604176 | 0,597642197 | -0,004442527 | 0,003310843 | 0,17965714 |
| Fasting plasma glucose (mmol/L) | -0,003927766 | 0,013349478 | 0,768787181 | -0,026383198 | 0,018809934 | 0,162655395 | -0,011449539 | 0,01088646 | 0,292926696 |
| Plasma glucose 2 hours after oral glucose tolerance test (mmol/L) | -0,014261756 | 0,01311988 | 0,277940234 | -0,035119701 | 0,017454072 | 0,045950722 | -0,021792137 | 0,01048744 | 0,037716058 |
| C-peptide (pmol/L) | -0,000119026 | 5,33E-05 | 0,026191328 | -8,44E-05 | 7,75E-05 | 0,277501056 | -0,000107912 | 4,39E-05 | 0,013949206 |
| Insulin (pmol/L) | -0,001432233 | 0,000495042 | 0,004094232 | -0,000614273 | 0,000548725 | 0,264613164 | -0,001065211 | 0,000367565 | 0,003755316 |
| Gestational diabetes mellitus 2013-criteria, 3 categories | -0,020348847 | 0,042815792 | 0,634940526 | -0,01173794 | 0,056180805 | 0,834762584 | -0,017185098 | 0,034053717 | 0,613806959 |
| Smoking, 4 categories | 0,022774209 | 0,015201504 | 0,135134039 | 0,042946058 | 0,065602834 | 0,513624013 | 0,023802128 | 0,01480912 | 0,10799702 |
| Total fat mass (%) | -0,009237303 | 0,001831425 | 7,93E-07 | -0,002440635 | 0,003436195 | 0,478609261 | -0,007733709 | 0,0016162 | 1,71E-06 |
| Truncal fat mass (%) | -0,013923073 | 0,003241384 | 2,36E-05 | -0,002222342 | 0,005253901 | 0,672893415 | -0,010697295 | 0,002758624 | 0,000105421 |
| HBA1C (%) | -0,049657048 | 0,063144081 | 0,43225097 | -0,067349345 | 0,081913687 | 0,412164785 | -0,056251617 | 0,050010083 | 0,260671456 |
| HOMA-B (%) | -0,000256913 | 0,000360875 | 0,477071765 | -0,00036882 | 0,000516151 | 0,475916622 | -0,000293656 | 0,000295756 | 0,320759924 |
| HOMA-IR (%) | -0,056426782 | 0,024182984 | 0,020293901 | -0,038855474 | 0,035110339 | 0,270088463 | -0,050773029 | 0,019915968 | 0,010791994 |
| Cholesterole (mmol/L) | -0,017757822 | 0,016543236 | 0,283937305 | 0,032500972 | 0,026410135 | 0,220236055 | -0,003594798 | 0,014019825 | 0,797635633 |
| HDL (mmol/L) | 0,07519901 | 0,043857269 | 0,087435498 | 0,035037636 | 0,062072099 | 0,573212457 | 0,06182585 | 0,035818647 | 0,084333028 |
| LDL (mmol/L) | -0,020876995 | 0,017855755 | 0,243253262 | 0,044866471 | 0,031246799 | 0,152966281 | -0,004693385 | 0,015503043 | 0,762088299 |
| Triglycerides (mmol/L) | -0,05527022 | 0,025486266 | 0,030888724 | -0,024226866 | 0,044367354 | 0,58577703 | -0,047568114 | 0,022099576 | 0,031362345 |
| Leptin (µg/L) | -0,065718995 | 0,01527367 | 2,29E-05 | -0,032911144 | 0,022829708 | 0,151347554 | -0,055574824 | 0,012694621 | 1,20E-05 |
| Gestational week | 0,0213993 | 0,014616245 | 0,14420974 | -0,029995815 | 0,022455683 | 0,183483827 | 0,00610484 | 0,012249897 | 0,618231393 |
| Mean systolic blood pressure (mmHg) | -0,004360972 | 0,001905413 | 0,022787802 | 0,001194284 | 0,003214351 | 0,710721452 | -0,002916482 | 0,001639074 | 0,075182789 |
| Mean diastolic blood pressure (mmHg) | -0,007110948 | 0,002559447 | 0,005807478 | 0,001345044 | 0,004005344 | 0,737452346 | -0,004659219 | 0,00215672 | 0,030747402 |
| CD4+ T-cells | -1,772024193 | 0,414986863 | 2,62E-05 | -2,11068917 | 0,646637732 | 0,001338142 | -1,870817186 | 0,349252054 | 8,48E-08 |
| CD8+ T-cells | -1,470502313 | 0,588116676 | 0,01293497 | -2,944846273 | 0,72555563 | 7,64E-05 | -2,055095646 | 0,456875888 | 6,85E-06 |
| B-cells | -3,617732497 | 1,51788033 | 0,017768931 | -4,204462462 | 1,618671345 | 0,010250629 | -3,892262749 | 1,107221852 | 0,000439189 |
| Neutrophiles | 1,057456794 | 0,240432417 | 1,51E-05 | 1,435895362 | 0,33380399 | 2,91E-05 | 1,186726501 | 0,195093235 | 1,18E-09 |
| Monocytes | -0,352035046 | 1,079658666 | 0,744602959 | 1,857895608 | 1,649795453 | 0,261762832 | 0,310612432 | 0,903404093 | 0,730978298 |
| NK-cells | -0,377138808 | 0,965978002 | 0,696499054 | -1,14666238 | 1,151744364 | 0,32092623 | -0,694913526 | 0,740124538 | 0,347774737 |
| Eosinophiles | 1,22E+15 | 2,79E+15 | 0,662669961 | -3,26E+15 | 4,61E+15 | 0,479669925 | 1,65047E+13 | 2,39E+15 | 0,994478506 |
| Parity | -0,003083518 | 0,036566672 | 0,932853003 | -0,010969984 | 0,056447739 | 0,846152626 | -0,00541473 | 0,030689951 | 0,859953538 |

HOMA-IR: Homeostatic model assessment for Insulin Resistance); HBA1c: Glycosylated haemoglobin A1c; HOMA-B: Homeostatic model assessment of beta-cell function; HDL: high density lipoprotein; LDL: low density lipoprotein; NK: natural killer.

# Supplementary table 11- Analysis of cardiometabolic parameters in association with DNA methylation of cg19758958 in linear regression models adjusted for age, smoking and estimated cell composition

|  | European ancestry | | | South Asian ancestry | | | Meta analysis | | |
| --- | --- | --- | --- | --- | --- | --- | --- | --- | --- |
| cg19758958 | **M-value** | **SE** | **P-value** | **M-value** | **SE** | **P-value** | **M-value** | **SE** | **P-value** |
| Age (whole years) | -0,003945364 | 0,002282255 | 0,084879092 | -0,002475732 | 0,003210621 | 0,441759859 | -0,003452036 | 0,001860169 | 0,063487105 |
| Fasting plasma glucose (mmol/L) | -0,008191898 | 0,007680224 | 0,286994337 | -0,025683911 | 0,00986523 | 0,010090857 | -0,014792822 | 0,006060238 | 0,014648115 |
| Plasma glucose 2 hours after oral glucose tolerance test (mmol/L) | -0,010974958 | 0,007596193 | 0,149613391 | -0,025887997 | 0,00909482 | 0,005023742 | -0,017103197 | 0,005830138 | 0,003350737 |
| C-peptide (pmol/L) | -1,52E-05 | 3,07E-05 | 0,621185366 | -4,83E-05 | 4,13E-05 | 0,24360979 | -2,70E-05 | 2,47E-05 | 0,273181362 |
| Insulin (pmol/L) | -0,000125166 | 0,000287366 | 0,66346735 | -0,000425478 | 0,000291839 | 0,146810143 | -0,000273003 | 0,000204762 | 0,182441717 |
| Gestational diabetes mellitus 2013-criteria, 3 categories | -0,029438164 | 0,024606316 | 0,232489001 | -0,064350915 | 0,029459453 | 0,030361943 | -0,043785695 | 0,018885185 | 0,020421028 |
| Smoking, 4 categories | 0,00749839 | 0,008775476 | 0,393519099 | 0,074886719 | 0,034445264 | 0,031141443 | 0,011605685 | 0,00850384 | 0,172329105 |
| Total fat mass (%) | -0,004199194 | 0,001059176 | 9,20E-05 | -0,005510171 | 0,001746305 | 0,001928467 | -0,004551765 | 0,000905619 | 5,00E-07 |
| Truncal fat mass (%) | -0,006620079 | 0,001863496 | 0,000443204 | -0,008517989 | 0,002665164 | 0,001690643 | -0,007243272 | 0,001527206 | 2,11E-06 |
| HBA1C (%) | -0,034250685 | 0,036390396 | 0,347361532 | -0,001052686 | 0,04366147 | 0,98079421 | -0,020642372 | 0,027954038 | 0,460247299 |
| HOMA-B (%) | 7,56E-06 | 0,000206852 | 0,970872409 | -0,000117511 | 0,000275532 | 0,670322316 | -3,75E-05 | 0,000165423 | 0,820557618 |
| HOMA-IR (%) | -0,006775002 | 0,013969908 | 0,628051929 | -0,022853177 | 0,018708109 | 0,223657721 | -0,012530812 | 0,01119347 | 0,262937472 |
| Cholesterole (mmol/L) | -0,013367275 | 0,00951346 | 0,16101865 | 0,002046354 | 0,014112201 | 0,884885299 | -0,008551202 | 0,007888399 | 0,278354835 |
| HDL (mmol/L) | 0,031438189 | 0,025312779 | 0,21520115 | -0,049528055 | 0,032820997 | 0,133225592 | 0,001240621 | 0,020044063 | 0,950646692 |
| LDL (mmol/L) | -0,013081174 | 0,010241564 | 0,202500194 | 0,019289395 | 0,016674213 | 0,249040771 | -0,00421419 | 0,008726863 | 0,62916775 |
| Triglycerides (mmol/L) | -0,040422019 | 0,014606099 | 0,005996752 | -0,013558284 | 0,023597445 | 0,566377136 | -0,032980801 | 0,012419491 | 0,007917502 |
| Leptin (µg/L) | -0,030108362 | 0,008904951 | 0,000817683 | -0,03557831 | 0,011786429 | 0,002950844 | -0,032096083 | 0,007105071 | 6,26E-06 |
| Gestational week | 0,015031435 | 0,008402123 | 0,074612884 | -0,001655849 | 0,01200906 | 0,890502842 | 0,009547368 | 0,006884423 | 0,165500563 |
| Mean systolic blood pressure (mmHg) | -0,001429739 | 0,001095539 | 0,192870916 | 0,00050875 | 0,00168391 | 0,762949855 | -0,000853246 | 0,000918299 | 0,352806576 |
| Mean diastolic blood pressure (mmHg) | -0,002772197 | 0,001473066 | 0,060811084 | -0,000982767 | 0,002097284 | 0,640000281 | -0,002181056 | 0,00120544 | 0,070397475 |
| CD4+ T-cells | -1,331147339 | 0,233866948 | 2,97E-08 | -1,03709397 | 0,345602857 | 0,003115969 | -1,23878869 | 0,193688142 | 1,60E-10 |
| CD8+ T-cells | -1,29187375 | 0,333992458 | 0,000134362 | -1,261562816 | 0,392727889 | 0,001586827 | -1,279152192 | 0,25442643 | 4,97E-07 |
| B-cells | -2,022978862 | 0,874527917 | 0,021379718 | -1,102722902 | 0,874381916 | 0,209058405 | -1,562774058 | 0,618332995 | 0,011491094 |
| Neutrophiles | 0,752260456 | 0,136106114 | 7,03E-08 | 0,51962258 | 0,182889395 | 0,005068662 | 0,669341367 | 0,109188166 | 8,78E-10 |
| Monocytes | 0,747558246 | 0,620338194 | 0,229112071 | 1,157401768 | 0,876284794 | 0,18841757 | 0,884381807 | 0,506310208 | 0,080686042 |
| NK-cells | -0,41409582 | 0,555882814 | 0,456889228 | 0,670042919 | 0,612247126 | 0,275394396 | 0,075783471 | 0,41155593 | 0,853904463 |
| Eosinophiles | -2,56E+15 | 1,60E+15 | 0,110676593 | -1,26E+15 | 2,45E+15 | 0,609283073 | -2,17E+15 | 1,34E+15 | 0,105259142 |
| Parity | 0,016984621 | 0,021034292 | 0,420027502 | -0,004311949 | 0,030027096 | 0,885992196 | 0,009974198 | 0,017227832 | 0,562617252 |

HOMA-IR: Homeostatic model assessment for Insulin Resistance); HBA1c: Glycosylated haemoglobin A1c; HOMA-B: Homeostatic model assessment of beta-cell function; HDL: high density lipoprotein; LDL: low density lipoprotein; NK: natural killer.

# Supplementary table 12- Analysis of cardiometabolic parameters in association with DNA methylation of cg10472537 in linear regression models adjusted for age, smoking and estimated cell composition

|  | European ancestry | | | South asian ancestry | | | Meta analysis | | |
| --- | --- | --- | --- | --- | --- | --- | --- | --- | --- |
| cg10472537 | **M-value** | **SE** | **P-value** | **M-value** | **SE** | **P-value** | **M-value** | **SE** | **P-value** |
| Age (whole years) | -0,003168349 | 0,003539351 | 0,371402547 | -0,008307025 | 0,004906331 | 0,092342228 | -0,004927197 | 0,002870419 | 0,0860628 |
| Fasting plasma glucose (mmol/L) | -0,024286906 | 0,011809013 | 0,04057956 | -0,013272571 | 0,015385669 | 0,389608677 | -0,020203717 | 0,009367787 | 0,031027283 |
| Plasma glucose 2 hours after oral glucose tolerance test (mmol/L) | -0,035972509 | 0,011770712 | 0,002454872 | -0,008889033 | 0,014373054 | 0,537191576 | -0,025100203 | 0,00910663 | 0,005846723 |
| C-peptide (pmol/L) | -0,000138011 | 4,71E-05 | 0,003646948 | -7,32E-05 | 6,32E-05 | 0,248125403 | -0,000114862 | 3,78E-05 | 0,00234955 |
| Insulin (pmol/L) | -0,001431129 | 0,000438654 | 0,001232026 | -0,000592571 | 0,000446972 | 0,186802382 | -0,001019725 | 0,000313075 | 0,001125454 |
| Gestational diabetes mellitus 2013-criteria, 3 categories | 0,03675718 | 0,038054333 | 0,334857958 | -0,065597616 | 0,045700977 | 0,153099016 | -0,005152737 | 0,029243529 | 0,860136097 |
| Smoking, 4 categories | -0,0142555 | 0,013552004 | 0,293678637 | -0,045237992 | 0,053648111 | 0,400332453 | -0,016113946 | 0,013139269 | 0,220049731 |
| Total fat mass (%) | -0,008488281 | 0,00162862 | 3,49E-07 | -0,006541479 | 0,002744977 | 0,018387956 | -0,007981404 | 0,001400648 | 1,21E-08 |
| Truncal fat mass (%) | -0,014637017 | 0,002855092 | 5,31E-07 | -0,009576466 | 0,004198894 | 0,023936997 | -0,013037028 | 0,002360992 | 3,35E-08 |
| HBA1C (%) | -0,017655484 | 0,056445481 | 0,754659578 | -0,028258435 | 0,067146941 | 0,674422833 | -0,022045717 | 0,04320725 | 0,609889014 |
| HOMA-B (%) | -0,000494619 | 0,000319946 | 0,123174985 | -0,000232853 | 0,000421359 | 0,58128833 | -0,000398889 | 0,000254812 | 0,117484044 |
| HOMA-IR (%) | -0,064554954 | 0,021378933 | 0,002749728 | -0,032042025 | 0,0286417 | 0,264926234 | -0,052921752 | 0,017132486 | 0,00200851 |
| Cholesterole (mmol/L) | -0,011282593 | 0,014734343 | 0,44443044 | 0,050216199 | 0,02135713 | 0,019905764 | 0,008549385 | 0,012128096 | 0,480857564 |
| HDL (mmol/L) | 0,044398673 | 0,039131246 | 0,257435871 | 0,097000148 | 0,050283514 | 0,055460068 | 0,064239182 | 0,030881832 | 0,037510773 |
| LDL (mmol/L) | -0,008380049 | 0,015989924 | 0,600608997 | 0,052364281 | 0,025517143 | 0,041767172 | 0,008747134 | 0,013549465 | 0,518557579 |
| Triglycerides (mmol/L) | -0,06297349 | 0,022565547 | 0,005593199 | -0,025021272 | 0,036294014 | 0,491548727 | -0,052392681 | 0,019163546 | 0,006257453 |
| Leptin (µg/L) | -0,036809535 | 0,013775759 | 0,007950934 | 0,013114539 | 0,018697026 | 0,484044975 | -0,019243646 | 0,011090538 | 0,082715843 |
| Gestational week | -0,009042267 | 0,013041521 | 0,488624536 | -0,009864328 | 0,018463641 | 0,593891695 | -0,009315888 | 0,01065223 | 0,381819792 |
| Mean systolic blood pressure (mmHg) | -0,003283514 | 0,001688852 | 0,052799386 | -0,002760698 | 0,002615287 | 0,292742117 | -0,003129656 | 0,001418749 | 0,027389114 |
| Mean diastolic blood pressure (mmHg) | -0,003008568 | 0,002285409 | 0,189033234 | -0,00044992 | 0,003269738 | 0,890728582 | -0,002168815 | 0,001873197 | 0,246939548 |
| CD4+ T-cells | 4,208269245 | 0,293455704 | 5,91E-36 | 4,033191898 | 0,445677847 | 4,16E-16 | 4,155320098 | 0,245095622 | 1,80E-64 |
| CD8+ T-cells | 5,722742043 | 0,414043556 | 5,00E-34 | 4,964993855 | 0,486894376 | 3,39E-19 | 5,404742264 | 0,315417449 | 8,11E-66 |
| B-cells | 9,653722417 | 1,245347306 | 1,38E-13 | 7,678992323 | 1,210830171 | 2,14E-09 | 8,638611587 | 0,868132654 | 2,50E-23 |
| Neutrophiles | -2,773852071 | 0,152644373 | 2,01E-50 | -2,638589799 | 0,201010892 | 2,55E-27 | -2,72438 | 0,121565769 | 3,09E-111 |
| Monocytes | -0,514165251 | 0,960424618 | 0,592799163 | -0,262009061 | 1,355408215 | 0,846959692 | -0,429878672 | 0,783636235 | 0,583301154 |
| NK-cells | 2,682288963 | 0,845848662 | 0,001674051 | 1,596533411 | 0,937233345 | 0,090389031 | 2,194911432 | 0,627934658 | 0,000473271 |
| Eosinophiles | 2,39E+15 | 2,48E+15 | 0,336150969 | 8,23E+15 | 3,72E+15 | 0,028380491 | 4,18E+15 | 2,06E+15 | 0,042620537 |
| Parity | -0,001272933 | 0,032538338 | 0,968819657 | -0,040323668 | 0,046098498 | 0,383008713 | -0,014258835 | 0,02658326 | 0,591693179 |

HOMA-IR: Homeostatic model assessment for Insulin Resistance); HBA1c: Glycosylated haemoglobin A1c; HOMA-B: Homeostatic model assessment of beta-cell function; HDL: high density lipoprotein; LDL: low density lipoprotein; NK: natural killer.

# Supplementary table 13- Analysis of cardiometabolic parameters in association with DNA methylation of cg16733643in linear regression models adjusted for age, smoking and estimated cell composition

|  | European ancestry | | | South asian ancestry | | | Meta analysis | | |
| --- | --- | --- | --- | --- | --- | --- | --- | --- | --- |
| cg16733643 | **M-value** | **SE** | **P-value** | **M-value** | **SE** | **P-value** | **M-value** | **SE** | **P-value** |
| Age (whole years) | -0.00127386686591979 | 0.00470914514082477 | 0.786955204807484 | -0.0134704299175908 | 0.0063756137248599 | 0.0362300837100813 | -0.00557905949547899 | 0.00378790918521651 | 0.140788820119316 |
| Fasting plasma glucose (mmol/L) | -0.0140585424361831 | 0.0159100080693307 | 0.37760889730836 | -0.0118230281796736 | 0.0199344899563407 | 0.553988184783023 | -0.0131886555843248 | 0.0124350572677061 | 0.288870485088144 |
| Plasma glucose 2 hours after oral glucose tolerance test (mmol/L) | -0.0139968453405952 | 0.0157120180175255 | 0.373778782616583 | -0.000789298991489334 | 0.0187801399376201 | 0.966533100023848 | -0.00855867778352734 | 0.0120507491061678 | 0.477567990256064 |
| C-peptide (pmol/L) | -0.000114558931744663 | 6.42749913110845e-05 | 0.0757246288583226 | -9.95467759884277e-05 | 8.23529031225581e-05 | 0.228623302630475 | -0.000108876005631933 | 5.06691230127426e-05 | 0.0316530840061091 |
| Insulin (pmol/L) | -0.00130677829447023 | 0.000598630919774083 | 0.0298280383413588 | -0.000587454366940728 | 0.00057566903813905 | 0.30912592177447 | -0.000933056279047929 | 0.000414939672299957 | 0.0245344373533385 |
| Gestational diabetes mellitus 2013-criteria, 3 categories | -0.073870723954629 | 0.0506981128449171 | 0.146146306829516 | -0.087324238075116 | 0.0596244646528609 | 0.145076553233936 | -0.079516018600948 | 0.0386233491673355 | 0.0395177011211362 |
| Smoking, 4 categories | -0.00946271443862644 | 0.0180626645201523 | 0.600748340772677 | 0.0471893503613606 | 0.0680456416281039 | 0.489043265057255 | -0.00573358924580591 | 0.0174580576678844 | 0.742593519117839 |
| Total fat mass (%) | -0.00448121317715537 | 0.00223043525503504 | 0.0454228879069052 | -0.00965594695019116 | 0.00348239377476562 | 0.00624441160500815 | -0.00598651189610843 | 0.00187821462591237 | 0.0014358700654871 |
| Truncal fat mass (%) | -0.00789293172158979 | 0.00390450728877701 | 0.04411944277592 | -0.0159794165697325 | 0.00529578310439142 | 0.00298420684745082 | -0.0107406675060167 | 0.00314267989674528 | 0.000631578122848123 |
| HBA1C (%) | 0.0780956801325528 | 0.0760152706841842 | 0.305088711280833 | -0.070583099414241 | 0.0873283090560663 | 0.420193510286967 | 0.0140044981388803 | 0.0573363044826405 | 0.807035776422407 |
| HOMA-B (%) | -0.000402613350570992 | 0.000428475792706007 | 0.348170557508975 | -0.000383143601491209 | 0.00054584333348465 | 0.483796478325629 | -0.000395190276913946 | 0.000337038659073943 | 0.240981551503718 |
| HOMA-IR (%) | -0.0553327540164962 | 0.0293774991385387 | 0.0606150922030321 | -0.0442586458710771 | 0.0372917656242291 | 0.237149874859838 | -0.0510920325476649 | 0.0230769516592369 | 0.0268297877706041 |
| Cholesterole (mmol/L) | -0.0027409185226359 | 0.0196457618688018 | 0.889135520917883 | 0.0271246735187929 | 0.0282168919868014 | 0.337912267953764 | 0.00700980130185085 | 0.0161228550793471 | 0.663726339002024 |
| HDL (mmol/L) | -0.000915929370302615 | 0.0525982424306938 | 0.986118203593095 | -0.0589613556075569 | 0.0651474834822383 | 0.366855664040276 | -0.0238216926337233 | 0.040924749148935 | 0.560509263270498 |
| LDL (mmol/L) | 0.00535090318714337 | 0.0211454766926743 | 0.800404350945411 | 0.0558066337049717 | 0.03387184222865 | 0.101490702004719 | 0.0195003420439854 | 0.0179371391380329 | 0.276970986132885 |
| Triglycerides (mmol/L) | -0.0264151522003126 | 0.0303047750153557 | 0.384098787784509 | -0.0175203217346382 | 0.0469956983755932 | 0.709804755912453 | -0.0238027779973039 | 0.0254687108935139 | 0.350000130791318 |
| Leptin (µg/L) | -0.0245237439672701 | 0.0184425410335206 | 0.184628470222458 | -0.024614415361392 | 0.0244427892700304 | 0.315514378049462 | -0.024556637062427 | 0.0147220398053165 | 0.0953120232194254 |
| Gestational week | 0.00529989212253212 | 0.0173270977352262 | 0.759914164143646 | -0.00767882884575902 | 0.024708578647037 | 0.756391286368164 | 0.00102142600171006 | 0.0141865196640132 | 0.942602099372945 |
| Mean systolic blood pressure (mmHg) | -0,003283514 | 0,001688852 | 0,052799386 | -0,002760698 | 0,002615287 | 0,292742117 | -0,003129656 | 0,001418749 | 0,027389114 |
| Mean diastolic blood pressure (mmHg) | -0,003008568 | 0,002285409 | 0,189033234 | -0,00044992 | 0,003269738 | 0,890728582 | -0,002168815 | 0,001873197 | 0,246939548 |
| CD4+ T-cells | -0.000197697609634946 | 0.00230320414236854 | 0.931654700272594 | -0.00424660867617633 | 0.00341578932549149 | 0.215710277280575 | -0.00146319342708322 | 0.0019096435715205 | 0.443549668743659 |
| CD8+ T-cells | -0.00221572968875122 | 0.00311198013032483 | 0.477023713578539 | -0.0009994931544176 | 0.00423974462436071 | 0.813950983182259 | -0.00178989427126984 | 0.00250871638059003 | 0.475554873205877 |
| B-cells | 4.75326314347199 | 0.425552847170908 | 1.92406767514042e-24 | 4.94641734860936 | 0.58240224411359 | 1.59540258829688e-14 | 4.82049385279953 | 0.343601303434879 | 1.0313059181365e-44 |
| Neutrophiles | 6.27009969431801 | 0.604448099459859 | 9.77407270071378e-22 | 5.23664930977762 | 0.702994493676767 | 6.28488695372409e-12 | 5.83082914040911 | 0.458324584725106 | 4.45992721209657e-37 |
| Monocytes | 14.53416693309 | 1.63015765724371 | 4.90659041260969e-17 | 8.43903207396021 | 1.59163050164349 | 3.90841375976163e-07 | 11.4137223863438 | 1.13882981413596 | 1.21608118570883e-23 |
| NK-cells | -3.36955829130012 | 0.220950472972863 | 3.03952632720741e-39 | -3.02034764910012 | 0.281619891868958 | 2.15552215290405e-20 | -3.23650355808295 | 0.173834013160417 | 2.28109759858105e-77 |
| Eosinophiles | 2.44742184785506 | 1.26685041040998 | 0.0543171490145514 | -0.378064093980555 | 1.79605075576457 | 0.833557703521489 | 1.50870845966263 | 1.03523402160757 | 0.145017084229793 |
| Parity | -0,0170943150671493 | 0,0434167551052666 | .694063801109495 | -0.0176003804990996 | 0.0598734335826673 | 0.769184468615595 | 0.00513784530655567 | 0.0351482825433213 | 0.883782227622543 |

HOMA-IR: Homeostatic model assessment for Insulin Resistance); HBA1c: Glycosylated haemoglobin A1c; HOMA-B: Homeostatic model assessment of beta-cell function; HDL: high density lipoprotein; LDL: low density lipoprotein; NK: natural killer.

# Supplementary table 14- Comparison of Houseman and FlowSorted.Blood.EPIC methods for cell type estimation in epigenome wide association study of body mass index in gestational week 28±2 and DNA methylation in peripheral white blood cells using a meta-analysis approach

|  |  |  |  |  | Houseman | | | FlowSorted.Blood.EPIC | | |
| --- | --- | --- | --- | --- | --- | --- | --- | --- | --- | --- |
| CpG-site | Relation to Island | Position | Chromosome | UCSC reference gene name | β | SE | P-value | β | SE | P-value |
| cg02786370* | OpenSea | 2747928 | chr4 | *TNIP2* | -0,0198 | 0,0031 | 2.124e-10 | -0.0173 | 0.0031 | 2.087e-08 |
| cg19758958* | OpenSea | 62319222 | chr11 | *Promoter region of AHNAC* | -0,0107 | 0,0018 | 1.116e-09 | -0.00962 | 0.00175 | 4.09e-08 |
| cg10472537* | OpenSea | 105348800 | chr2 |  | -0,0111 | 0,002 | 2.05e-08 | -0.0106 | 0.00198 | 7.691e-08 |
| cg16444328 | OpenSea | 171279429 | chr5 |  | -0,0082 | 0,0015 | 3.763e-08 | -0.00724 | 0.00151 | 1.52e-06 |
| cg24911837 | N_Shore | 65227864 | chr7 | *CCT6P1* | -0,0131 | 0,0024 | 3.963e-08 | -0.0116 | 0.00239 | 1.117-06 |
| cg23191724* | OpenSea | 70267556 | chr11 | *CTTN* | -0,0178 | 0,0033 | 8.47e-08 | -0.0176 | 0.00331 | 1.021e-07 |
| cg01270753* | OpenSea | 101944336 | chr9 |  | -0,017 | 0,0032 | 1.008e-07 | -0.0141 | 0.00320 | 1.305 e-05 |
| cg26050822 | OpenSea | 30320193 | chrX |  | -0,0241 | 0,0046 | 1.249e-07 | -0.0237 | 0.00459 | 2.233e-07 |
| cg08298765 | OpenSea | 123983480 | chr9 |  | -0,0072 | 0,0014 | 1.948e-07 | -0.00528 | 0.00141 | 0.000176 |
| cg20887442 | OpenSea | 71725424 | chr11 | *NUMA1* | -0,0082 | 0,0016 | 2.533e-07 | -0.00678 | 0.00166 | 4.589e-05 |
| cg05551937 | S_Shore | 57776726 | chr4 | *REST* | 0,0119 | 0,0023 | 2.899e-07 | 0.0123 | 0.00232 | 1.0218e-07 |
| cg02702424* | OpenSea | 30654871 | chr3 | *TGFBR2* | -0,0093 | 0,0018 | 3.516e-07 | -0.00749 | 0.00196 | 0.00013 |
| cg03340148 | OpenSea | 11971431 | chr16 | *GSPT1* | -0,0093 | 0,0018 | 3.67e-07 | -0.00732 | 0.00182 | 5.797e-05 |
| cg12737110 | Island | 219861126 | chr2 |  | -0,0132 | 0,0026 | 3.944e-07 | -0.012 | 0.00269 | 8.465e-06 |
| cg06222414 | N_Shelf | 44636919 | chr20 | *MMP9* | -0,015 | 0,003 | 5.26e-07 | -0.0128 | 0.00302 | 2.204e-05 |
| cg10251538* | OpenSea | 108800886 | chr3 | *MORC1* | -0,0133 | 0,0027 | 6.148e-07 | -0.0106 | 0.00276 | 0.000118 |
| cg18972123 | OpenSea | 54532059 | chr10 | *MBL2* | -0,0092 | 0,0018 | 6.59e-07 | -0.00805 | 0.00185 | 1.399e-05 |
| cg20715780 | OpenSea | 186814701 | chr1 | *PLA2G4A* | -0,0079 | 0,0016 | 6.637e-07 | -0.00732 | 0.00156 | 2.85e-06 |
| cg22851864* | OpenSea | 5688470 | chr10 | *ASB13* | -0,0326 | 0,0066 | 6.661e-07 | -0.0279 | 0.00657 | 2.18e-05 |
| cg27179379 | OpenSea | 63988325 | chr1 | *EFCAB7* | -0,0293 | 0,0059 | 7.065e-07 | -0.0285 | 0.0059 | 1.405e-06 |
| cg11254085 | Island | 109744679 | chr2 | *SH3RF3* | 0,0107 | 0,0022 | 7.112e-07 | 0.01 | 0.00213 | 2.576e-06 |
| cg22528063 | OpenSea | 127114914 | chr9 | *LOC100129034* | -0,0066 | 0,0013 | 7.425e-07 | -0.00542 | 0.001331 | 4.743e-05 |
| cg12404216 | S_Shore | 46743199 | chr3 | *TMIE* | -0,008 | 0,0016 | 8.929e-07 | -0.00708 | 0.00165 | 1.776e-05 |
| cg15239145 | S_Shore | 128285392 | chr2 | *IWS1* | -0,0101 | 0,0021 | 9.059e-07 | -0.00959 | 0.00205 | 2.779e-06 |
| cg06088069* | S_Shore | 75895604 | chr14 | *JDP2* | -0,0063 | 0,0013 | 9.366e-07 | -0.00598 | 0.00129 | 3.313e-06 |
| cg02693156 | OpenSea | 9903454 | chr12 |  | -0,0477 | 0,0098 | 1.072e-06 | -0.0426 | 0.00994 | 1.826e-05 |
| cg12404181 | OpenSea | 5688515 | chr10 | *ASB13* | -0,0279 | 0,0057 | 1.133e-06 | -0.023 | 0.00572 | 5.736e-05 |
| cg27560175 | OpenSea | 48118501 | chr20 |  | -0,02 | 0,0041 | 1.179e-06 | -0.0194 | 0.00411 | 2.243e-06 |
| cg05271333 | OpenSea | 43084407 | chr6 | *PTK7* | -0,0118 | 0,0024 | 1.208e-06 | -0.0106 | 0.0025 | 2.406e-05 |
| cg13823169* | N_Shelf | 139776893 | chr9 |  | -0,0085 | 0,0018 | 1.369e-06 | -0.00742 | 0.00178 | 2.941e-05 |
| cg20786944 | OpenSea | 29606019 | chr21 |  | -0,0077 | 0,0016 | 1.426e-06 | -0.00621 | 0.0016 | 0.000107 |
| cg02543993* | OpenSea | 5736195 | chr7 | *RNF216* | -0,0201 | 0,0042 | 1.635e-06 | -0.0182 | 0.00418 | 1.368e-05 |
| cg15198407 | OpenSea | 80841737 | chr10 | *ZMIZ1* | -0,0084 | 0,0018 | 1.734e-06 | -0.0066 | 0.00183 | 0.000311 |
| cg05630316* | OpenSea | 56830679 | chr8 | *LYN* | -0,0084 | 0,0018 | 1.77e-06 | -0.00748 | 0.00179 | 2.943e-05 |
| cg01885635 | N_Shore | 40566085 | chr3 | *ZNF621* | -0,0079 | 0,0016 | 1.828e-06 | -0.00683 | 0.00163 | 2.846e-05 |
| cg23223006 | S_Shore | 44203109 | chrX | *EFHC2* | -0,0174 | 0,0037 | 2.073e-06 | -0.0152 | 0.00373 | 4.857e-05 |
| cg06988896* | S_Shelf | 78426368 | chr15 |  | -0,0079 | 0,0017 | 2.162e-06 | -0.00652 | 0.00171 | 0.000133 |
| cg25691233 | OpenSea | 5721828 | chr7 | *RNF216* | 0,0107 | 0,0023 | 2.198e-06 | 0.0107 | 0.0022 | 1.328e-06 |
| cg25364972 | OpenSea | 217075573 | chr2 |  | -0,0103 | 0,0022 | 2.21e-06 | -0.00932 | 0.00226 | 3.715e-05 |

# Supplementary figure 1- Flow chart of the study
